# Multiple Requirements of PLK1 during Mouse Oocyte Maturation

**DOI:** 10.1371/journal.pone.0116783

**Published:** 2015-02-06

**Authors:** Petr Solc, Tomoya S. Kitajima, Shuhei Yoshida, Adela Brzakova, Masako Kaido, Vladimir Baran, Alexandra Mayer, Pavlina Samalova, Jan Motlik, Jan Ellenberg

**Affiliations:** 1 Institute of Animal Physiology and Genetics, Libechov, Czech Republic; 2 Cell Biology and Biophysics Unit, European Molecular Biology Laboratory, Heidelberg, Germany; 3 Institute of Animal Physiology, Kosice, Slovakia; 4 Laboratory for Chromosome Segregation, RIKEN Center for Developmental Biology, Kobe, Japan; National Cancer Institute, NIH, UNITED STATES

## Abstract

Polo-like kinase 1 (PLK1) orchestrates multiple events of cell division. Although PLK1 function has been intensively studied in centriole-containing and rapidly cycling somatic cells, much less is known about its function in the meiotic divisions of mammalian oocytes, which arrest for a long period of time in prophase before meiotic resumption and lack centrioles for spindle assembly. Here, using specific small molecule inhibition combined with live mouse oocyte imaging, we comprehensively characterize meiotic PLK1’s functions. We show that PLK1 becomes activated at meiotic resumption on microtubule organizing centers (MTOCs) and later at kinetochores. PLK1 is required for efficient meiotic resumption by promoting nuclear envelope breakdown. PLK1 is also needed to recruit centrosomal proteins to acentriolar MTOCs to promote normal spindle formation, as well as for stable kinetochore-microtubule attachment. Consequently, PLK1 inhibition leads to metaphase I arrest with misaligned chromosomes activating the spindle assembly checkpoint (SAC). Unlike in mitosis, the metaphase I arrest is not bypassed by the inactivation of the SAC. We show that PLK1 is required for the full activation of the anaphase promoting complex/cyclosome (APC/C) by promoting the degradation of the APC/C inhibitor EMI1 and is therefore essential for entry into anaphase I. Moreover, our data suggest that PLK1 is required for proper chromosome segregation and the maintenance of chromosome condensation during the meiosis I-II transition, independently of the APC/C. Thus, our results define the meiotic roles of PLK1 in oocytes and reveal interesting differential requirements of PLK1 between mitosis and oocyte meiosis in mammals.

## Introduction

Polo-like kinase 1 (PLK1) plays a variety of roles in mitotic cell division[1–3]. PLK1 controls the timing of mitotic entry[4], centrosome maturation[5], chromosome cohesion[6], kinetochore-microtubule attachment[4,7], and cytokinesis[8–12]. Although much information about the mitotic roles of PLK1 has been accumulated, our understanding of PLK1 function during meiosis in mammalian oocytes remains relatively poor. It has been reported that in mammalian oocytes, PLK1 is involved in resumption of meiosis[13–15], spindle formation[15,16], and RhoA-mediated cytokinesis[17]. However, how PLK1 controls resumption of meiosis and spindle formation, as well as in other meiotic processes such as kinetochore-microtubule attachment, APC/C activation, and meiosis I—meiosis II transition, are largely unknown in oocytes.

One unique feature of the oocyte is that it undergoes a long prophase arrest before entering M-phase of meiosis I. Resumption of meiosis is characterized by two visible, tightly coupled events: nuclear envelope breakdown (NEBD) and chromosome condensation. The resumption depends on the activity of CDK1, which is positively regulated by the phosphatases CDC25A/B[18,19]. PLK1 controls meiotic resumption in oocytes by participation in CDK1 autoamplification loop[14]. In somatic cells, PLK1 controls the activity of CDK1 by phosphorylating cyclin B[20] and multiple regulators, such as CDC25B[21], CDC25C[22,23] and WEE1[24], although it is not absolutely essential for mitotic entry[4,25]. Moreover, after DNA damage and subsequent arrest at the G2 phase in somatic cells, PLK1 promotes G2-checkpoint recovery[26,27], which is very similar in many aspects to the resumption of meiosis I[28]. However, a direct requirement of PLK1 independent of CDK1 activity for the resumption of meiosis I in oocytes has not been experimentally demonstrated.

Another unique feature of the oocyte is that it lacks centriole-containing centrosomes. In oocytes, instead of centrosomes, there are many acentriolar MTOCs throughout the cytoplasm, which self-assemble into an acentriolar bipolar spindle[29]. It is known that in somatic cells, PLK1 localizes to centrosomes[30] and promotes microtubule nucleation by recruiting the γ-tubulin complex. In mouse oocytes, PLK1 localizes to MTOCs[15,16] and microinjection of PLK1 antibody disturbs normal bipolar spindle formation[15]. However, it is unknown whether PLK1 regulates the maturation of acentriolar MTOCs and how PLK1 impacts the kinetics of spindle formation and bipolarization.

As the spindle forms, condensed chromosomes gradually congress towards the equatorial plane of the forming spindle, resulting in a belt-like spatial arrangement of chromosomes, the prometaphase belt[31]. After the prometaphase belt is formed, microtubules begin the process of chromosome biorientation by attaching to kinetochores. In contrast to somatic cells, the most prominent kinetochore fibers that can be detected in oocytes with electron microscopy form at the very late stage of metaphase I[32], suggesting an oocyte-specific regulation of kinetochore-microtubule attachments. Although it is known that in somatic cells, PLK1 stabilizes kinetochore-microtubule attachments during prometaphase and maintains correct attachment during metaphase[4,7,25,33,34], it remains unclear whether PLK1 plays a similar role in kinetochore-microtubule attachment during oocyte meiosis.

Kinetochore-microtubule attachment silences the SAC, a surveillance mechanism that inhibits anaphase onset[35]. Once the SAC is silenced, the APC/C becomes activated. The APC/C mediates the degradation of securin and cyclin B to trigger anaphase. In mitosis, PLK1 inhibition leads to a failure in APC/C activation because a defect in kinetochore-microtubule attachment activates the SAC[4,7,25]. In addition, PLK1 has been speculated to regulate APC/C activity. PLK1 phosphorylates the APC/C inhibitor EMI1, mediating its degradation during prometaphase[36,37]. PLK1 also directly phosphorylates the APC/C, which is implicated in regulating APC/C activity[38–40]. However, neither the EMI1 degradation nor APC/C phosphorylation is essential for anaphase entry in mammalian somatic cells because the mitotic arrest caused by PLK1 perturbation can be released by the inactivation of the SAC alone[4,7,25,40]. It is unknown whether PLK1 is involved in these pathways that activate the APC/C during oocyte meiosis I, and if so, whether they are essential for anaphase I onset.

In this study, we comprehensively investigate the multiple functions of PLK1 in mouse oocyte meiosis by using specific and acute temporal pharmacological inhibition, combined with live imaging and quantitative immunostaining. We demonstrate that PLK1 promotes nuclear envelope permeabilization during NEBD independently of CDK1 activity. Similarly as in mitosis, PLK1 recruits centrosomal proteins to acentriolar MTOCs, promotes efficient spindle formation and kinetochore-microtubule attachment, and thus is required for satisfying the SAC. In contrast to mitosis, SAC silencing is not sufficient for anaphase I entry in PLK1-inhibited oocytes because PLK1 is essential for APC/C activation through at least two pathways independently of the SAC satisfaction. In addition, our data suggest meiotic-specific requirements of PLK1 for chromosome segregation and the maintenance of the condensed state of chromosomes during the meiosis I—meiosis II transition.

## Results

### PLK1 dynamically changes its localization during meiotic maturation

**Fig 1:**
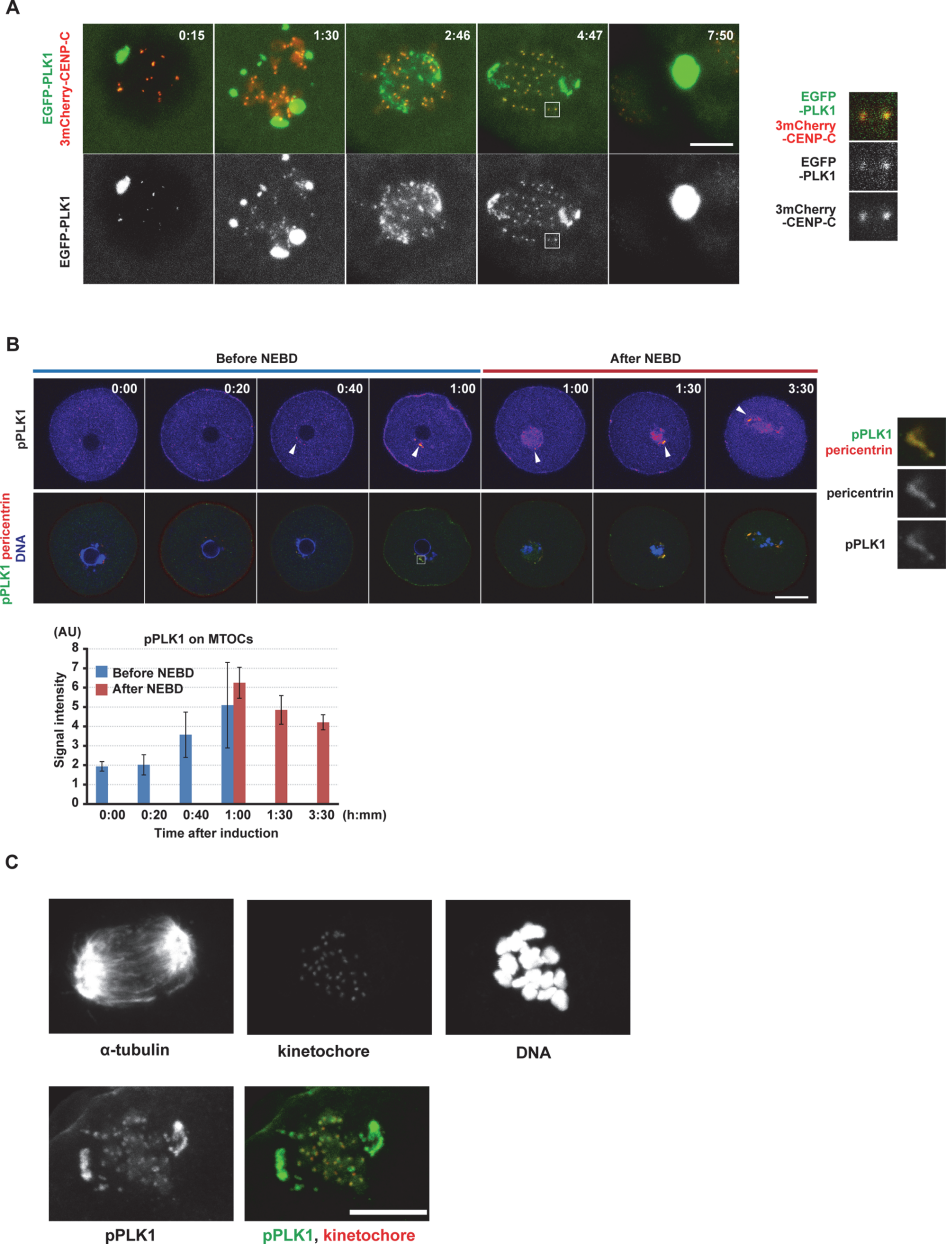
PLK1 localizes to MTOCs and kinetochores and becomes activated around NEBD. (A) Imaging of meiosis I in oocytes expressing EGFP-PLK1 (green) and 3mCherry-CENP-C (kinetochores, red). Maximum intensity z-projection images are shown. At 7:50, the saturated signal locates at the spindle midzone, as shown in S1A Fig. Time after induction of meiotic resumption (h:mm). Scale bar = 10 μm. Insets show magnified images on kinetochores. Also see S1 Movie. (B) Immunostaining of active PLK1 phosphorylated on T210 (pPLK1) during meiosis I. Pictures represent single selected confocal sections through MTOCs for pPLK1 (fire-pseudocolored or green) and pericentrin (MTOCs, red) and maximum intensity z-projection for DAPI (DNA, blue). Arrowheads indicate pPLK1 signals on MTOCs. Time after induction of meiotic resumption (h:mm). Quantification of MTOC-associated pPLK1 signals (n = 11, 5, 7, 5, 5, 9, 17 oocytes). Averages with the 95% confidence intervals are shown. Scale bar = 20 μm. Insets show magnified images on MTOCs. (C) Localization of pPLK1 on kinetochores in metaphase I oocytes. Oocytes stained for pPLK1 (green), kinetochores (CREST, red), acetylated α-tubulin, and DAPI (DNA). Scale bar = 10 μm.

It has been reported that PLK1 localizes uniformly in the cytoplasm at the germinal vesicle (GV) stage, at MTOCs and kinetochores from NEBD to metaphase I, and at the spindle midzone at anaphase I in mouse oocytes [14,15,41]. However, the changes in PLK1 localization in live oocytes have not been quantitatively analyzed. To address this question, we recorded 4D datasets of EGFP-PLK1 localization in live oocytes. EGFP-PLK1 exhibited punctate signals throughout the cytoplasm at the GV stage (S1A Fig.), and localized to the spindle poles from prometaphase to metaphase I (Fig. 1A, S1 Movie). The EGFP-PLK1 also co-localized with the kinetochore marker 3mCherry-CENP-C (Fig. 1A, S1 Movie). The localizations at MTOCs and kinetochores were further confirmed by immunostaining of endogenous PLK1 (Fig. 1B, C). At anaphase I, EGFP-PLK1 disappeared from the spindle poles and kinetochores and relocated to the spindle midzone (Fig. 1A, S1A Fig., S1 Movie) with approximately 12-fold enrichment above the levels on the earlier kinetochores (S1B Fig.). Thus, these results confirm that PLK1 dynamically changes its localization between three key cell division structures during meiosis I in mouse oocytes.

### PLK1 becomes activated during the resumption of meiosis

Previous biochemical analysis has indicated that PLK1 activity increases before resumption of meiosis and persists during meiotic maturation[14]. To examine the local PLK1 activity in individual oocytes in a time course, we used quantitative immunofluorescence staining for the phosphorylation of T210 (pPLK1), which specifically labels the activated form of PLK1[26]. The pPLK1 signals began to be detected 20 minutes before NEBD (40 minutes after the induction of meiotic resumption) colocalizing with the MTOC marker pericentrin, and reached its maximum level at the time of NEBD (Fig. 1B). From prometaphase to metaphase I, pPLK1 was highly concentrated on MTOCs and kinetochores (Fig. 1B,C), which is consistent with the results of EGFP-PLK1. These data demonstrate that PLK1 becomes activated around NEBD at MTOCs during the resumption of meiosis and, later, also at kinetochores.

### PLK1 localizations on MTOCs and kinetochores are differentially regulated by its own activity

The temporal difference in PLK1 localizations between MTOCs and kinetochores prompted us to ask whether these localizations are differentially controlled by PLK1’s own activity. To this end, we treated oocytes with BI2536, a small molecule inhibitor known to specifically inhibit PLK1 in somatic cells [25] and in oocytes [42] as well as in chemical genetics studies [43,44]. We tested the concentrations of 20–200 nM BI2536, and found that the 100 nM concentration maximally reduced the phosphorylation of BUBR1 at T669 (S1C Fig.), the residue that corresponds to the known PLK1 target T680 of human BUBR1 on mitotic kinetochores [45]. Because this concentration had no detectable effect on the histone H3 S28 phosphorylation (S1D Fig.), which depends on Aurora kinases in oocytes [46], and is shown to specifically inhibit PLK1 in somatic cells[25], we used the 100 nM concentration in all experiments to inhibit PLK1 in this study. In BI2536-treated oocytes, the EGFP-PLK1 localization on MTOCs dramatically decreased (S1E Fig.), whereas the level on kinetochores significantly increased (S1E Fig.). Thus, these results suggest that in oocytes, the PLK1 localizations at MTOCs and kinetochores are differentially regulated by its own activity.

### PLK1 is required for the timing of nuclear envelope permeabilization

**Fig 2:**
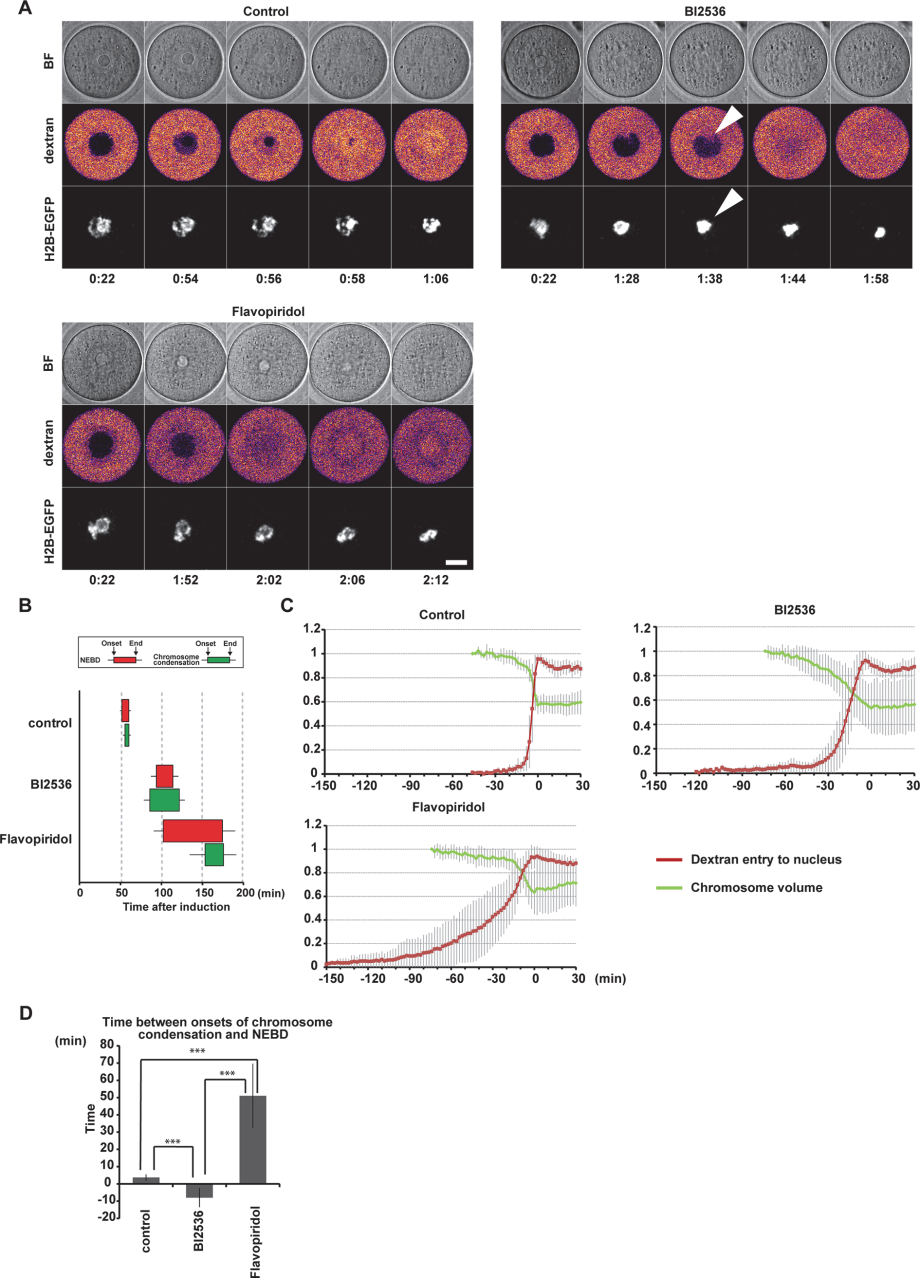
PLK1 promotes NEBD during meiotic resumption. (A) Imaging of H2B-EGFP-expressing oocytes microinjected with 70kDa-dextran-TAMRA in control, 100 nM BI2536 and 1 μM flavopiridol medium. Pictures represent single section from bright field (BF), single confocal section of 70kDa-dextran-TAMRA signals (fire-pseudocolored) and maximum intensity z-projection for H2B-EGFP (gray). Arrowheads indicate condensed chromosomes in the intact nucleus, specifically observed in BI2536-treated oocytes. Time after induction of meiotic resumption (h:mm). Scale bar = 20 μm. Also see S2 Movie. (B) Onsets and ends of chromosome condensation (green) and NEBD (red) in control, BI2536, and flavopiridol oocytes were defined according to S2 Fig. by measuring chromosome volume and the nuclear entry of 70 kDa dextran-TAMRA. The left and right sides of the box indicates the mean timings of the onset and end of the process, respectively, with the 95% confidence intervals (n = 16, 17, 15). (C) Kinetics of chromosome condensation (green) and 70 kDa-dextran-TAMRA nuclear entry (red) in control, BI2536 and flavopiridol oocytes. Curves represents mean values with s.d. (n = 16, 17, 15). Time at the point when chromosomes reached minimum volume was defined as t = 0. Chromosome volumes were normalized to 1 at -46 minutes in control and at -74 minutes in BI2536 and flavoporidol groups. Nuclear dextran signals were scaled between 0 and 1, according to the global minimum and maximum. (D) Time delay between onsets of chromosome condensation and dextran nuclear entry (delay = t2 - t1, according to S2 Fig.). Averages with the 95% confidence intervals are shown (n = 16, 17, 15; ***p<0.001).

The activation of PLK1 before NEBD (Fig. 1 B,C) is consistent with its known role in CDK1 autoamplification during resumption of meiosis[14,47]. The resumption of meiosis is associated with two clearly visible changes in the oocytes: NEBD and chromosome condensation. To explore the possibility that PLK1 plays a role in these processes, we microinjected fluorescent 70 kDa-dextran into the cytoplasm of H2B-EGFP-expressing oocytes and treated them with BI2536 immediately after the induction of meiotic resumption. To analyze the progression of chromosome condensation, we reconstructed the H2B-EGFP chromosome signals in 3D and measured its volume. To analyze the permeabilization of nuclear membranes as the first step of NEBD, we measured the entry of 70 kDa-dextran into the nucleus (Fig. 2A-C, S2 Fig. and S2 Movie). In BI2536-treated oocytes, both NEBD and chromosome condensation were significantly delayed (Fig. 2B), indicating that PLK1 is required for promoting these processes. Moreover, we noticed that the onset of NEBD was more affected than that of chromosome condensation by the BI2536 treatment (Fig. 2B), which was manifested by the presence of condensed chromosomes inside of the intact nucleus (Fig. 2A, BI2536 arrowheads). Chromosome condensation was triggered prematurely 8.0 ± 5.5 minutes before the onset of dextran entry when PLK1 was inhibited, while chromosome condensation normally started 3.8 ± 1.7 minutes after the onset of dextran entry in control oocytes (Fig. 2B-D). Thus, these results suggest that PLK1 is required for timely initiation of NEBD followed by the onset of chromosome condensation.

Because PLK1 is involved in an autoamplification loop activating CDK1[20,21], it was possible that the effects on the timing of NEBD and chromosome condensation by PLK1 inhibition are solely consequences of impaired CDK1 activity. To address this possibility, we used partial pharmacological CDK1 inhibition by 1 μM flavopiridol [48]. As anticipated, flavopiridol-treated oocytes were overall delayed in chromosome condensation and NEBD onsets oocytes (Fig. 2A, B). However, in contrast to PLK1 inhibition, CDK1 inhibition delayed the onset of chromosome condensation much more than that of NEBD (Fig. 2B-D). Live imaging of lamin B1-EGFP further demonstrated distinct effects of CDK1 versus PLK1 inhibition. While lamin disassembly was delayed by flavopirodol, it was not affected by BI2536 (S3A–B Fig.). These results suggest that effect of PLK1 on NEBD and chromosome condensation cannot be explained only by the known role of PLK1 in CDK1 activation but PLK1 has probably additional functions in regulatiing these processes. Collectively, our results suggest that PLK1 promotes timely initiation of NEBD.

### PLK1 promotes the formation of acentriolar bipolar spindles

**Fig 3:**
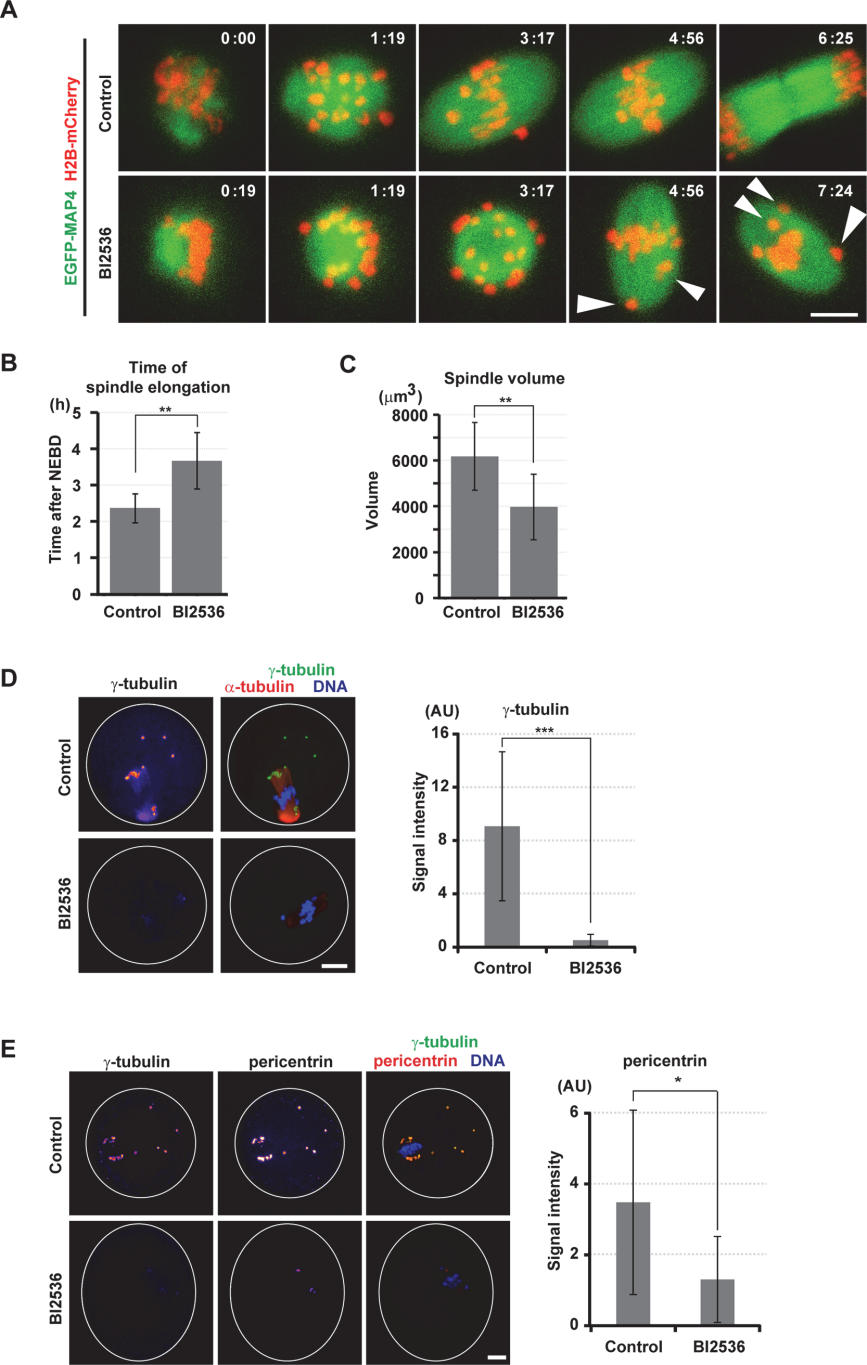
PLK1 is required for spindle formation with acentriolar MTOCs. (A) Imaging of meiosis I in oocytes expressing EGFP-MAP4 (microtubules, green) and H2B-mCherry (chromosomes, red) in the presence of DMSO (control) or 100 nM BI2536. Maximum intensity z-projection images are shown. Arrowheads indicate misaligned chromosomes. Time after NEBD (h:mm). Scale bar = 10 μm. Also see S3 Movie. (B) The EGFP-MAP4 signals were reconstructed in 3D (see S4A Fig.) and the time of spindle elongation was determined. Averages and s.d. are shown (n = 8, 17. **p < 0.01). (C) The volume of the spindle at 6 hours after NEBD was measured after 3D reconstruction of EGFP-MAP4 signals. Average and s.d (n = 8, 17. **p < 0.01). (D) Control and BI2536-treated oocytes at metaphase I were stained either for γ-tubulin (green) and acetylated α-tubulin (red) or (E) for γ-tubulin (green) and pericentrin (red). DNA was stained with DAPI (blue). Maximum z-projections of entire confocal stacks are shown. Scale bar = 10 μm. Quantifications of the spindle pole-associated integrated signals of γ-tubulin and pericentrin. Averages with the 95% confidence intervals are shown (n = 6, 7, 4, 4. ***p < 0.002, *p < 0.05).

In mitosis, PLK1 activity is required for centrosomal microtubule nucleation and the establishment of spindle bipolarity[4,5,7,25]. To explore the role of PLK1 in acentriolar spindle formation in oocytes, we imaged the microtubule marker EGFP-MAP4[29] in live oocytes treated with BI2536 (Fig. 3A, S3 Movie). In control oocytes, after NEBD, microtubules formed an apolar ball-like spindle, the microtubule ball, which elongated into a bipolar spindle 2.4 ± 0.4 hours after NEBD (Fig. 3A,B). In the BI2536-treated oocytes, however, the time of spindle elongation was significantly delayed to 3.7 ± 0.8 hours after NEBD. Furthermore, the sizes of the formed microtubule ball and elongated bipolar spindle were significantly smaller in the BI2536-treated oocytes (Fig. 3C, S4A–B Fig.). Thus, PLK1 is required for efficient bipolarization and establishment of the proper size of the acentriolar spindle.

### PLK1 promotes the recruitment of γ-tubulin and pericentrin to MTOCs

In somatic cells, PLK1 facilitates the recruitment of centrosomal material including γ-tubulin and pericentrin[5,49–52]. We found that in BI2536-treated oocytes, the poles of the metaphase I spindle contained a markedly lower amount of γ-tubulin and considerably reduced pericentrin (Fig. 3D,E). Moreover, whereas control oocytes contained several cytoplasmic MTOCs that were not associated with the spindle poles, such MTOCs were never observed in the BI2536-treated oocytes (Fig. 3D,E). Collectively, these data indicate that the defects in spindle assembly kinetics in PLK1-inhibited oocytes are at least partly a consequence of strongly compromised MTOCs.

### Prometaphase belt formation in PLK1-inhibited oocytes

**Fig 4:**
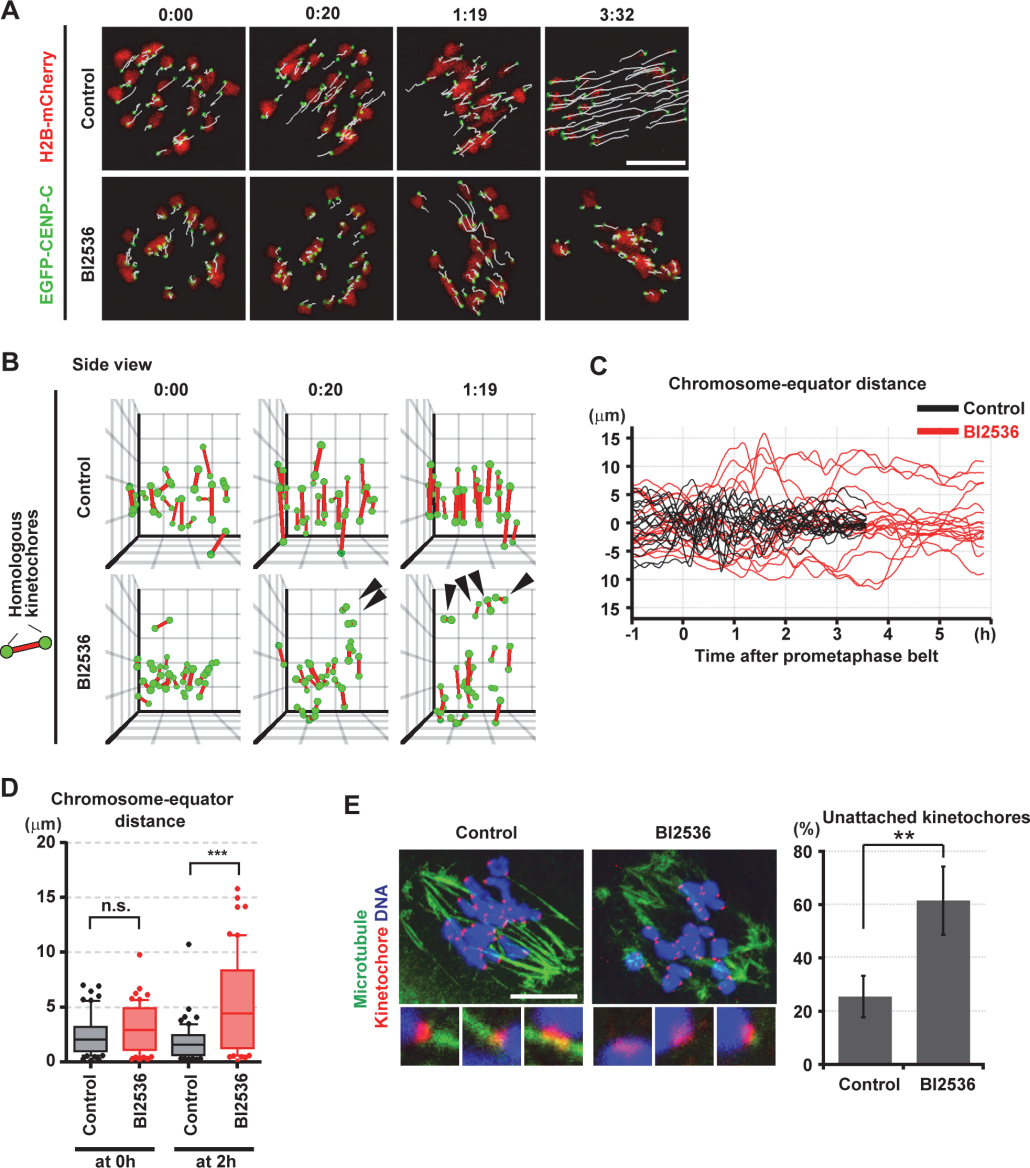
PLK1 is required for chromosome alignment. (A) Imaging of meiosis I in oocytes expressing EGFP-CENP-C (kinetochores, green) and H2B-mCherry (chromosomes, red) in the presence of DMSO (control) or 100 nM BI2536. Maximum intensity z-projection images are shown. White lines indicate kinetochore tracks over 5 timepoints. Time after NEBD (h:mm). Scale bar = 10 μm. Also see S4 Movie. (B) Kinetochore positions were determined from (A) and shown in the 3D plot as green spheres. Red bars connect homologous kinetochores. The view along the chromosome distribution equator (side view) is shown. Black arrowheads indicate misaligned chromosomes. Time after prometaphase belt formation (h:mm). The unit of the grid is 5 μm. (C) Chromosome positions along the estimated spindle axis were plotted for all twenty chromosomes of a single oocyte cultured in the presence of DMSO (control, black) or 100 nM BI2536 (red). (D) Distances between chromosomes and the equator at 0 and 2 hours after the prometaphase belt formation were potted. The box indicates 10–90 percentile (n = 60, 60, 60, 60 from three oocytes for each condition). ***p < 0.0001. (E) Oocytes 4 hours after NEBD were briefly treated with a cold buffer and fixed for immunostaining of microtubules (blue) and kinetochores (red). 100 nM BI2536 was added at 2 hours after NEBD. DNA was stained with Hoechst33342 (blue). Insets show magnified images of kinetochore-microtubule attachments. Scale bar = 10 μm. Average and s.d. of the population of unattached kinetochores are shown (n = 5, 5. **p < 0.01).

Although the MTOC function is impaired in BI2536-treated oocytes, a bipolar spindle is still formed. Nevertheless, the BI2536-treated oocytes showed chromosome misalignment at metaphase I (Fig. 3A,D). To address how chromosomes fail to align in these oocytes, we analyzed chromosome dynamics in detail by high resolution live imaging and the complete tracking of kinetochores[31] (Fig. 4A, S4 Movie). We observed a slight but not significant delay in the prometaphase belt formation (S5A–B Fig.) (2.5 ± 0.2 hours after NEBD in control v.s. 2.8 ± 0.5 hours in BI2536-treated oocytes, p = 0.81), although the arrangement was somewhat distorted (S5A Fig.). In this arrangement, most of the chromosomes were nevertheless located around the spindle equator (Fig. 4B, 0:00), exhibiting a mean chromosome-equator distance that was comparable to that in the control oocytes (2.4 ± 1.8 μm in the control vs. 3.1 ± 2.1 μm in the BI2536-treated, p = 0.053) (Fig. 4D, 0 hour). Thus, the defect in prometaphase belt formation could contribute to, but does not fully account for, the chromosome misalignment that was observed at metaphase I in the PLK1-inhibited oocytes.

### PLK1 is required for proper kinetochore-microtubule attachment

In oocytes, kinetochore-microtubule attachment begins to be detected after the establishment of the prometaphase belt[31,32]. In mitosis, PLK1 is required for stable kinetochore-microtubule attachment[4,7,25,34]. To assess whether PLK1 plays a similar role in oocytes, we analyzed chromosome movements after prometaphase belt formation. In control oocytes, the majority of the chromosomes began biorientation 20 minutes after the establishment of the prometaphase belt, oscillating within 8 μm around the spindle equator, and the chromosome alignment was maintained until the onset of anaphase I (Fig. 4B,C). However, in BI2536-treated oocytes, the chromosomes moved towards the spindle poles during biorientation attempts, and their positions reached a maximum of 16 μm away from the spindle equator (Fig. 4B,C). The mean chromosome-equator distance became markedly different between the control and BI2536-treated oocytes 2 hours after establishment of the prometaphase belt (1.8 ± 1.6 μm in the control vs. 5.2 ± 4.4 μm in the BI2536-treated oocytes, p < 0.0001) (Fig. 4D). These misaligned chromosomes frequently remained at the polar region of the spindle until late metaphase I (Fig. 4C). These data indicate that the majority of misaligned chromosomes observed in the BI2536-treated metaphase I oocytes were generated after the completion of prometaphase belt formation.

To examine whether this defect is related to kinetochore-microtubule attachments, we immunostained stable microtubules after cold treatment. In BI2536-treated oocytes, the population of unattached kinetochores was significantly increased (Fig. 4E), indicating that PLK1 activity is required for stable kinetochore-microtubule attachment. Consistently, we found that the distance between homologous kinetochores at metaphase I was significantly shorter in BI2536-treated oocytes (S5C Fig.). In human somatic cells, one of the PLK1 targets critical for kinetochore function is the residue T680 of BUBR1[45], which corresponds to T669 of mouse BUBR1. We found that the BUBR1 phosphorylation level at T669 was significantly decreased in BI2536-treated oocytes (S1C Fig.). Collectively, our data suggest that PLK1 phosphorylates BUBR1 and promotes stable kinetochore-microtubule attachment.

### The APC/C is not activated in PLK1-inhibited oocytes

**Fig 5:**
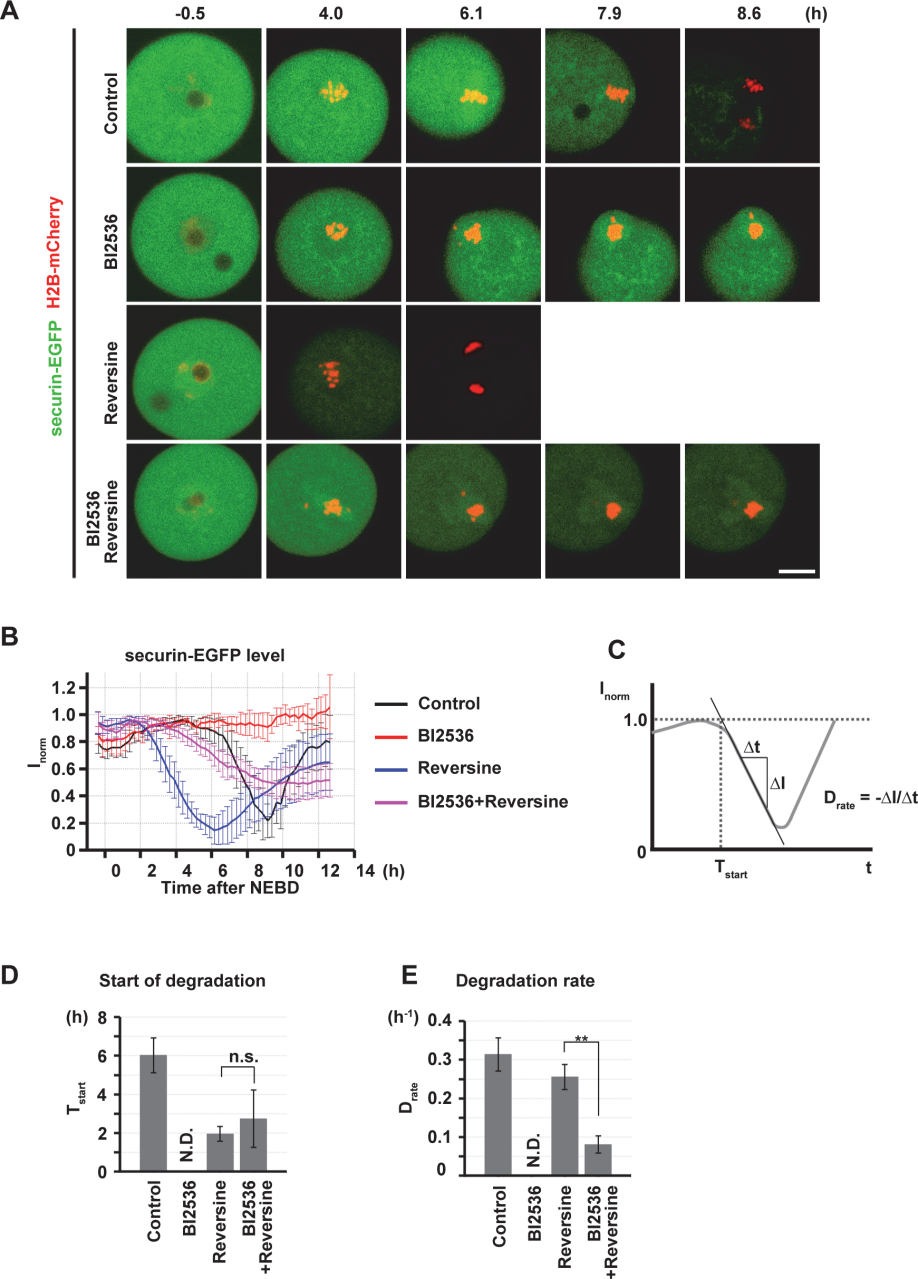
PLK1 activates the APC/C through multiple pathways. (A) Imaging of oocytes expressing securin-EGFP (green) and H2B-mCherry (chromosomes, red) in the presence of DMSO (control), 100 nM BI2536 and/or 1 μM reversine. Maximum intensity z-projection images are shown. Time after NEBD (h). Scale bar = 10 μm. Also see S5 Movie. (B) Normalized intensities of cytoplasmic securin-EGFP signals (I_norm_) were plotted. Average and s.d. are shown (n = 6, 4, 12, 15). (C-E) A line was fitted to the decrease of I_norm_ (C). Time for the start of securin-EGFP degradation (T_start_) was defined as the time when the fitted line reaches the I_norm_ value 1.0. The degradation rate (D_rate_) was defined as the negative value of the slope of the fitted line. Averages with s.d. of T_start_ and D_rate_ are shown in (D) and (E), respectively (n = 6, 12, 15. **p < 0.01).

In somatic cells, PLK1-inhibited cells arrest at mitosis with misaligned chromosomes because the APC/C is not activated due to the prolonged activity of the SAC[4,7,25]. Consistent with this, we found that BI2536-treated oocytes failed to enter anaphase I with misaligned chromosomes (Figs. 3 and 4). To confirm that this arrest is due to the absence of APC/C activation, we monitored the change in APC/C activity using the established marker securin-EGFP[53]. In control oocytes, the securin-EGFP level began to decline at approximately 6 hours after NEBD, indicating that the APC/C became activated, and the securin-EGFP level reached the minimum of approximately 20% at 9 hour, which coincided with the onset of anaphase I (Fig. 5A,B, S5 Movie). However, in BI2536-treated oocytes, the securin-EGFP level was virtually constant for 12 hours after NEBD, indicating that the APC/C is not activated in the absence of PLK1 activity.

### PLK1 is required for full APC/C activation, independently of satisfying the SAC

To test whether the block of APC/C activation in PLK1-inhibited oocytes is SAC-dependent, we inhibited MPS1, an essential kinase for the SAC function, using the specific inhibitor reversine[54]. As anticipated, the oocytes that were treated with reversine alone prematurely triggered securin-EGFP destruction and entered anaphase I with defective chromosome segregation (Fig. 5A-D, S5 Movie), consistent with the known function of MPS1 in mouse oocytes[55]. In the oocytes that were treated with reversine in addition to BI2536, a decrease in the securin-EGFP level was observed (Fig. 5A,B), indicating that the activation of the APC/C occurred. Thus, the block of APC/C activation in BI2536-treated oocytes depends, at least partly, on the SAC activity. Importantly, BI2536 did not delay the initiation of APC/C activation in the presence of reversine (Fig. 5B,D), confirming that our condition of reversine treatment largely, if not completely, abolished the SAC function. Nevertheless, BI2536 treatment significantly slowed the kinetics of the securin degradation in the presence of reversine (Fig. 5B,E). In these double-treated oocytes, approximately 50% of the securin-EGFP remained after the degradation, and none of the oocytes entered anaphase I (n = 15). Taken together, our results strongly suggest that PLK1 is required for the APC/C to be fully activated, independently of its primary function in satisfying the SAC. This PLK1 function is essential for entry into anaphase I in oocytes, unlike in somatic cells[4,7,25,40].

### PLK1 is required for degradation of the APC/C inhibitor EMI1

**Fig 6:**
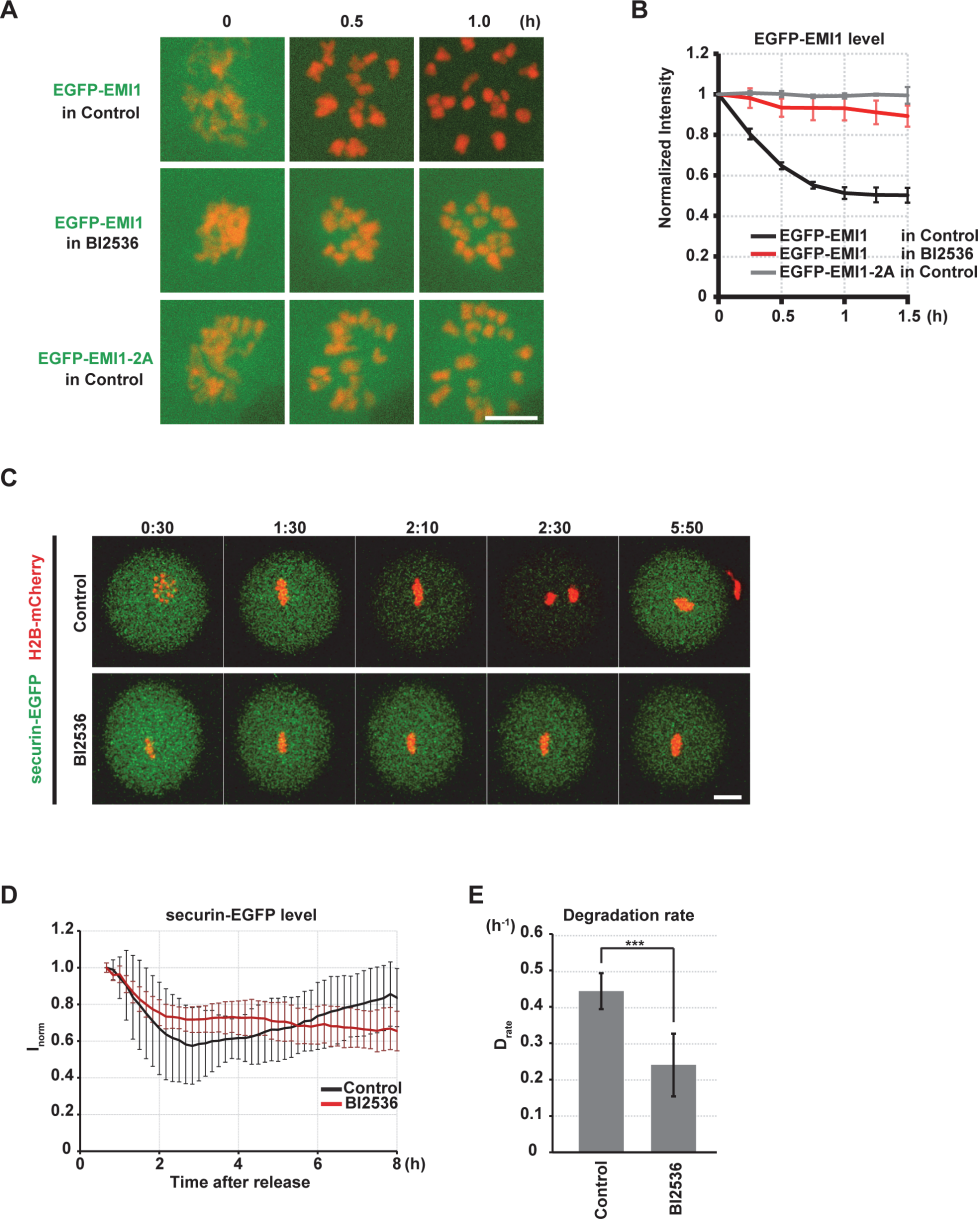
PLK1 is required for EMI1 destruction and full APC/C activation. (A) Imaging of oocytes expressing EGFP-EMI1 or EGFP-EMI1–2A (green) and H2B-mCherry (red) in the presence of DMSO (control) or 100 nM BI2536. Scale bar = 10 μm. Time after NEBD (h). (B) Cytoplasmic EGFP-EMI1 signals were measured and normalized. Average and s.d. are shown (n = 7, 5, 3). Time after NEBD (h). (C) Imaging of securin-EGFP (green) and H2B-mCherry (red) after DMSO (control, top) or 100 nM BI2536 (bottom) was added at the time of metaphase I (6.5 hours after the induction of meiotic resumption). Note that in the control oocyte at 0:30, the metaphase plate is viewed from the top of the spindle. Time after BI2536 addition (h:mm). Scale bar = 20 μm. Also see S6 Movie. (D) Quantification of securin-EGFP destruction. Values were normalized to 1 at the time when imaging was started (n = 11, 18). Time is relative to BI2536 addition (h). (E) Degradation rate of securin-EGFP in oocytes with BI2536 addition during metaphase I. Average and s.d. are shown.***p < 0.001.

Next, we sought to identify PLK1-dependent pathways that activate the APC/C independently of satisfying the SAC. To address whether PLK1 is required for degradation of the APC/C inhibitor EMI1, we monitored EGFP-EMI1 degradation in oocytes[56]. In the BI2536-treated oocytes, EGFP-EMI1 degradation was severely compromised (Fig. 6A,B), indicating that PLK1 is also required for EMI1 degradation in meiosis. EMI1-2A, which harbors mutations that substitute 124S and 128S—the residues corresponding to the PLK1-dependent phosphorylation sites 145S and 149S of human EMI1—with alanines, was less efficiently phosphorylated by PLK1 than wild-type EMI1 *in vitro* (S6 Fig.). In oocytes, EGFP-EMI1-2A failed to be degraded after NEBD (Fig. 6A,B). Thus, these data suggest that PLK1 promotes EMI1 degradation by phosphorylating EMI1 to activate the APC/C.

### PLK1 likely activates the APC/C through a third pathway

We further explored the possibility that PLK1 has more pathways that activate the APC/C. To address this, we added BI2536 to the oocytes at late metaphase I, during which all chromosomes had been established stable alignment. We reasoned that the oocytes at this stage should already have degraded EMI1 and satisfied the SAC, thus allowing us to target a pathway independent of EMI1 degradation and SAC satisfaction. In this condition, we found that the degradation of securin-EGFP was significantly slower than that in the control oocytes (Fig. 6C,D,E, S6 Movie). It is unlikely that this is due to reactivation of the SAC because chromosome alignment was stably maintained (Fig. 6C, S6 Movie). The BI2536-treated oocytes were unable to reach the same minimal securin-EGFP level as controls until 8 hours after the addition of BI2536 (Fig. 6D), and they remained arrested at metaphase I without any signs of chromosome segregation (Fig. 6C). Thus, these data suggest the presence of a third pathway through which PLK1 activates the APC/C.

### PLK1 is required for chromosome segregation and maintenance of the condensed state of chromosomes as well as for cytokinesis

**Fig 7:**
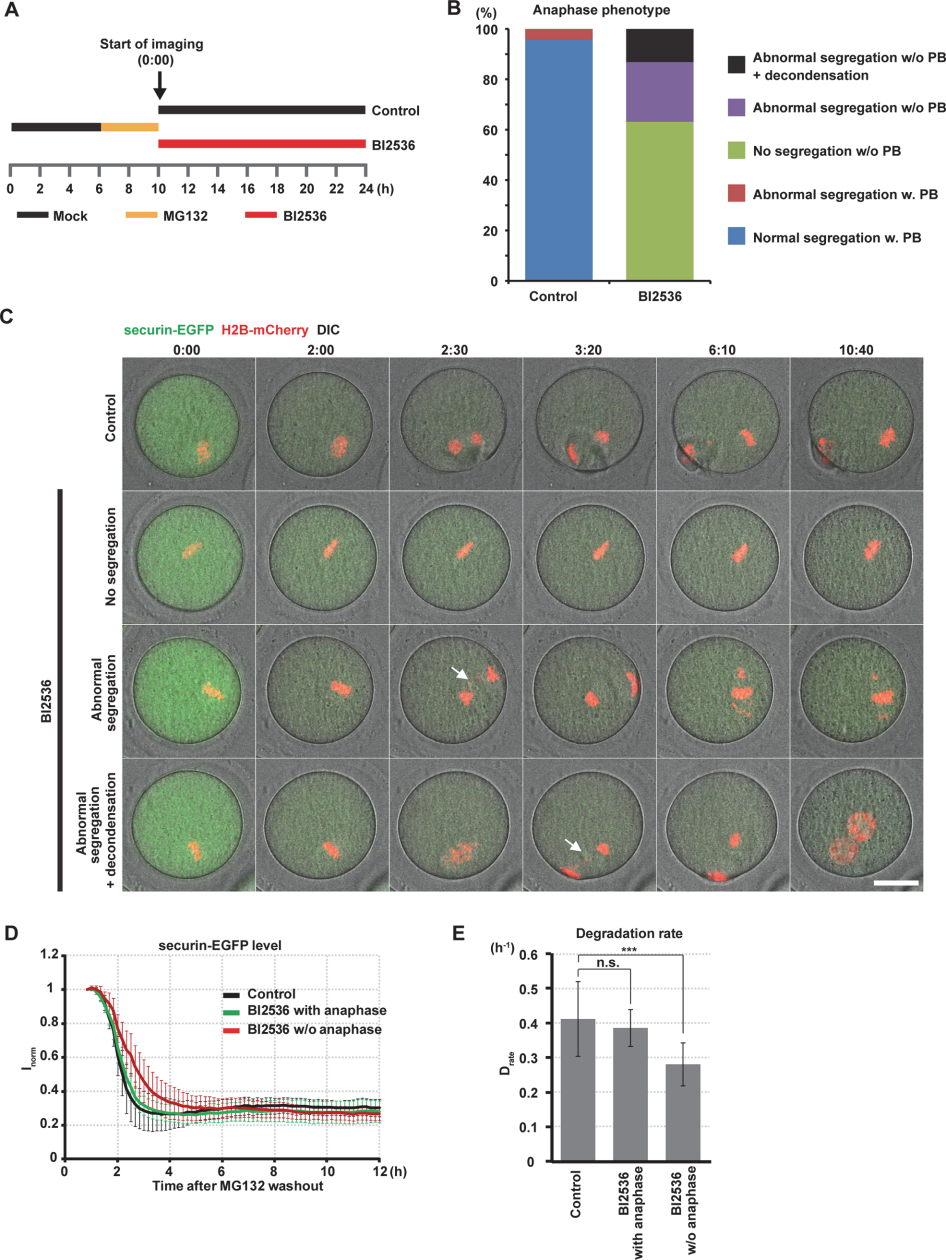
PLK1 is required for chromosome segregation, first polar body extrusion, and maintenance of the condensed state of chromosomes. (A) Experimental scheme. Oocytes were cultured for 6 hours in control medium, and then MG132 was added. The oocytes were incubated for 4 hours to arrest oocytes at the late metaphase I. After release from MG132, 100 nM BI2536 was added and oocytes were imaged. (B) Anaphase phenotypes after MG132 release in control and BI2536-treated oocytes. PB = the first polar body. (C) Imaging of securin-EGFP (green) and H2B-mCherry (red) after DMSO (control, top) or 100 nM BI2536 (lower panels) was added at the time of the MG132 washout. Each phenotype from Fig. 6B is shown on a representative image sequence. Arrows indicate lagging chromosomes. Note that none of the BI2536-treated oocytes undergoing abnormal chromosome segregation extruded the first polar body. Time after MG132 washout (h:mm). Scale bar = 30 μm. Also see S7 Movie. (D) Quantification of securin-EGFP destruction. Values were normalized to 1 when the imaging was started. Time relative to MG132 washout (h). The ‘BI2536 with anaphase’ curve represents BI2536-treated oocytes that underwent abnormal chromosome segregation either with or without DNA decondensation (3^rd^ and 4^th^ rows in Fig. 7C). The ‘BI2536 w/o anaphase’ curve represents BI2536-treated oocytes that did not undergo chromosome segregation (2^nd^ row in Fig. 7C). Average and s.d. are shown (n = 23, 40). (E) Degradation rate of securin-EGFP calculated from (D). Average and s.d. are shown. ***p < 0.001.

Although our data revealed that PLK1 is required for APC/C activation through multiple pathways, there can be additional PLK1 functions that occur later than APC/C activation that have not been uncovered by the above-mentioned approaches due to the strong metaphase I arrest of the BI2536-treated oocytes. To solve this problem, we arrested oocytes at the metaphase/anaphase I transition by adding of the proteasome inhibitor MG132 after 6 hours of meiotic maturation when EMI1 is already depleted[56,57]. Thereafter, we released them into anaphase I by washout (Fig. 7A). We reasoned that if APC/C substrates are largely ubiquitinated during the MG132 arrest, the oocytes after MG132 washout could have a reduced requirement of PLK1 for APC/C activation to enter anaphase I. Indeed, when we added BI2536 immediately after washing out MG132, 37% of the oocytes exhibited a comparable kinetics of securin degradation to control oocytes and entered anaphase I at a normal timing (Fig. 7B,C,D,E, S7 Movie). The intact degradation of APC/C substrates was further supported by the observation of the normal kinetics of cyclin B degradation in the same experimental condition (S7B–D Fig.). In these oocytes, however, we detected strong chromosome segregation problems, such as many lagging chromosomes and chromosome bridges, whereas only 4% of the controls exhibited segregation problems with only one lagging chromosome (Fig. 7B,C). None of these BI2536-treated oocytes extruded the first polar body (Fig. 7B), which is in good agreement with the known role of PLK1 in cytokinesis in mitosis and meiosis[8–12,17]. Moreover, in 13% of the BI2536-treated oocytes, chromosomes that had failed segregation partially decondensed at metaphase II (Fig. 7B), forming two pronucleus-like structures (Fig. 7C). Collectively, our data suggest that PLK1 is required for chromosome segregation independently of APC/C activation and maintenance of the condensed state of chromosomes during the meiosis I-meiosis II transition.

## Discussion

### PLK1 localizations on MTOCs and kinetochores are differentially regulated by its own activity

The polo-box domain (PBD) of PLK1 is required for the localization of PLK1 to centrosomes and unattached kinetochores in mitotic cells [58]. PLK1 phosphorylates kinetochore protein PBIP1 (also known as CENP-U) at T78, creating a binding site for the PBD and thus targeting PLK1 to kinetochores [59]. Inhibition of PLK1 by BI2536 in mitotic cells prevents PLK1 recruitment to centrosomes and kinetochores [25]. As expected, BI2536 treatment decreased the EGFP-PLK1 level on MTOCs in oocytes. However, inhibition of PLK1 activity increased the level of EGFP-PLK1 at kinetochores (S1E Fig.). Thus, it is possible that PLK1 is recruited to kinetochores through unknown pathways in oocytes, in addition to the PLK1 activity-dependent pathways used in mitotic cells.

### PLK1 ensures the correct timing of NEBD and chromosome condensation

Chromosome condensation and NEBD are clearly visible hallmarks of the mitotic entry of somatic cells and the meiotic resumption of oocytes. In somatic cells, chromosome condensation and NEBD are promoted by PLK1. NEBD is a step-wise process that involves nuclear pore disassembly and microtubule-dependent mechanical tearing of the nuclear envelope, followed by depolymerization of the nuclear lamina[60,61]. Nuclear pore disassembly is dependent on the phosphorylation of nuclear pore proteins[62]. Recently, many nuclear pore proteins were determined to be phosphorylated by PLK1[63]. Microtubule-dependent forces on the nuclear envelope are mainly generated by the dynein-dynactin complex[64], whose activity at NEBD depends on PLK1-mediated phosphorylation of the p150Glued subunit[65]. To facilitate chromosome condensation, PLK1 is recruited to chromosome axes through binding to the CAP-D3 condensin II subunit that is phosphorylated by CDK1 and phosphorylates many other subunits of the condensin II complex[66].

In oocytes, we confirmed that PLK1 promotes both chromosome condensation and NEBD. Unlike in somatic cells, PLK1 significantly contributes to nuclear envelope permeabilization as a first step of NEBD and ensures that it starts before chromosome condensation (Fig. 2), suggesting an oocyte-specific function of PLK1. This timing function of PLK1 is not solely a consequence of impaired CDK1-cyclin B activation because PLK1 inhibition but not partial CDK1 inhibition inverted the order of NEBD and chromosome condensation (Fig. 2). Unlike in somatic cells, microtubules are not involved in NEBD in starfish [60] and mouse oocytes (data not shown). Because lamin disassembly was not affected by PLK1 inhibition (S3 Fig.), it is likely that PLK1 plays a major role in promoting NEBD by phosphorylation of nuclear pore proteins. In contrast, PLK1 likely plays a relatively minor role in chromosome condensation, as PLK1 is not absolutely essential for chromosome condensation in mitosis [4,7]. Thus, PLK1 could support the timely onset of NEBD just before chromosome condensation by contributing to each process at different levels, in coordination with PLK1-independent pathways.

### PLK1 promotes the meiotic spindle assembly

Our data revealed that PLK1 plays a crucial role in acentriolar spindle formation in oocytes (Fig. 3, S4 Fig.). Similar to what occurs in centriolar somatic cells, PLK1 recruits centrosomal materials such as γ-tubulin and pericentrin to acentriolar MTOCs, promoting meiotic spindle formation. It is worth noting that, unlike in somatic cells[5] and acentriolar Xenopus egg extracts[67], PLK1 function was not absolutely required for spindle bipolarization in mouse oocytes (Fig. 3A). It is unlikely that this was due to the partial inhibition of PLK1 activity because bipolar spindle formation was observed at even higher BI2536 concentrations such as 1–2 μM (data not shown).

### PLK1 activates the APC/C through multiple pathways

Our data strongly suggest that PLK1 regulates APC/C activity through multiple pathways in oocytes. First, PLK1 promotes proper kinetochore-microtubule attachment (Fig. 4, S5 Fig.), which satisfies the SAC and thereby enables APC/C activation (Fig. 5). Second, PLK1 phosphorylates the APC/C-inhibitor EMI1 promoting its degradation (Fig. 6A,B, S6 Fig.). Future studies are needed to confirm the existence of the third pathway (Fig. 6C,D), which could involve APC/C phosphorylation by PLK1[38–40]. Importantly, our data highlighted a clear difference between somatic cells and oocytes in the requirement of the PLK1-dependent APC/C activation. Somatic cells do not require PLK1 to enter anaphase if the SAC is abrogated[4,7,25], whereas oocytes require it (Figs. 5 and 6C,D). This difference could stem from the large volume of oocytes. Oocytes could have larger amounts of EMI1, securin, and cyclin B than somatic cells, and thus more APC/C activity would be needed to degrade them.

### PLK1 functions in the meiosis I—II transition

Our results suggested that PLK1 promotes chromosome segregation at anaphase I independently of APC/C activation (Fig. 7). This could involve the PLK1-dependent phosphorylation of cohesin REC8, which allows REC8 to be cleaved by separase *in vitro*[68]. Consistent with this concept, a significant fraction (approximately 60%) of BI2536-treated oocytes after MG132 washout remained arrested at metaphase I, despite the observation that they degraded securin (Fig. 7D) and cyclin B (S7C Fig.) to the same minimal level as control oocytes. Our results also suggested that PLK1 maintains the condensed state of chromosomes at metaphase II (Fig. 7C). Because chromosome decondensation at metaphase II in BI2536-treated oocytes was rescued by introducing exogenous cyclin B-EGFP (S7A,B Fig.), PLK1 could prevent chromosome decondensation by ensuring a minimal essential CDK1-cyclin B activity at metaphase II.

## Conclusions

Taken together, our results define the meiotic roles of PLK1 in mammalian oocytes and reveal different requirements between mitosis and oocyte meiosis. PLK1 in oocytes not only promotes resumption of meiosis similar to what occurs in mitotic entry but also controls the onset of nuclear envelope permeabilization. Similar to what occurs in centriolar mitosis, PLK1 in oocytes facilitates meiotic spindle formation by recruiting centrosomal proteins to acentriolar MTOCs, and it promotes stable kinetochore-microtubule attachment, leading to the SAC satisfaction. In addition, in contrast to mitosis, it is essential in oocytes that PLK1 fully activates the APC/C through multiple pathways, one of which is EMI1 degradation, for anaphase I entry. Lastly, PLK1 ensures correct chromosome segregation, mediates first polar body extrusion, and prevents chromosome decondensation during the meiosis I—meiosis II transition in oocytes.

## Materials and Methods

### Animal use ethical statement

Using mice in this study was approved by the Departmental Expert Committee for the Approval of Projects of Experiments on Animals of the Academy of Sciences of the Czech Republic (for IAPG CAS), by The Animal Care and Use Committee of RIKEN CDB and by EMBL Institutional Animal Care and Use Committee. Animal welfare was under control of local committees. Mice were housed in a temperature-controlled room with proper 12/12 darkness-light cycles, fed with a regular ad libitum diet.

### Culture of oocytes and microinjection

For oocyte isolation 8–12-week-old CD1, BDF1, FVB or H2B-EGFP [69] mice were stimulated by 5 IU of pregnant mare's serum gonadotropin (PMSG) 46 hours before experiment and killed by cervical dislocation. Oocytes were collected into the M2 medium and cultured either in Opti-MEM medium (Life Technologies) supplemented with 10% FCS in 5% CO_2_ atmosphere or in M2 at 37°C. Meiotic maturation was prevented by 2.5 μM milrinone (Sigma-Aldrich). BI2536 (ChemieTek or Axon Medchem BV), reversine (Cayman Chemical or Merck Millipore), MG132 (Calbiochem or Sigma-Aldrich) and flavopiridol (Sigma Aldrich) were used at 100 nM, 1 μM, 1 μM, and 1 μM respectively.

We microinjected oocytes[70] with 2 pl mEGFP-PLK1, 1 pl EGFP-CENP-C[31], 4pl 3mCherry-CENP-C[31], 0.2 pl H2B-mCherry[31], 1.5 pl EGFP-MAP4[29], 1 pl securin-EGFP[71], 0.5 pl cyclin B1-EGFP[72], and 1 pl mEGFP-EMI1, from 1 μg/μl mRNA stocks. For nuclear envelope permeability measurement, H2B-EGFP oocytes were microinjected with 5 pl of 2 mg/ml 70-kDa dextran conjugated with tetramethylrhodamine (TAMRA) (Sigma Aldrich, D-1819).

### Live confocal microscopy and image analysis

Time-lapse image acquisitions in Figs. 1, 3, 4, 5, 6A, and S1 Fig. were performed using a Zeiss LSM710 equipped with a 40x C-Apochromat 1.2 W Corr M27 objective lens (Carl Zeiss). Using a 3D multi-location tracking macro[73], we recorded the subvolume centered around chromosomes at every time point. Kinetochore tracking, determination of the chromosome distribution equator, and estimation of the spindle axis with EGFP-CENP-C, and measurement of spindle volume and aspect ratio with EGFP-MAP4 were performed as described[31]. The time of prometaphase belt formation in Fig. 4 was defined as the time when the number of chromosomes that were located in the prometaphase belt region reached a maximum (S5B Fig.).

Time-lapse image acquisitions in Figs. 2, 6C, 7, S3 and S7 Figs. were performed using Leica TCS SP5 with an HCX PL Apo Lambda Blue 40x 1.25 oil objective. Image analysis was performed using Fiji software [74]. In Fig. 2, H2B-EGFP-expressing oocytes microinjected with 70-kDa-dextran-TAMRA were sequentially irradiated with 3% of the 488 nm and 561 nm laser and scanned. 3D+t image datasets of individual oocytes were registered using H2B-EGFP signal by the Correct 3D Drift plug-in in Fiji. Chromosome volume was measured from 3D reconstructed images when H2B signal was intensity-thresholded. Dextran in the area of the nucleus was measured from the single confocal section through the center of the nucleus. Identifications of the onset and end of AF, dextran influx, and chromosome condensation were done using defined thresholds (S2 Fig.).

### Immunostaining

Immunostaining was conducted as previously described in Figs. 1 and 3 [75] and Fig. 4 and S1 Fig. [31]. pT210 PLK1 was detected by rabbit polyclonal (1: 100; Santa Cruz Biotechnology), kinetochores by human CREST serum (1:100; Europa Bioproducts), α-tubulin by mouse monoclonal (1:500; Sigma), BUBR1 by sheep polyclonal (1:100; Abcam), pT669 BUBR1 by rabbit polyclonal [76]at 1:500 with the peptide LIKKLSPIIEESREATHSSGF at 100 μg/ml, histone H3 by rabbit polyclonal (1:500; Abcam), and pS28 H3 by rat monoclonal (1:500; Abcam) antibodies. For stable KT-MT attachment detection, oocytes were cultured in ice-cold M2 medium for 10 minutes before fixation.

### In vitro phosphorylation assay and Western blot

Recombinant proteins of GST-EMI1 (1–211 a.a., wild-type) and GST-EMI1–2A (S124A, S128A) were purified from BL21 (DE3) pLysS E. coli cells (Promega) with Glutathione-sepharose beads (GE). His-PLK1 was bound to Ni sepharose 6 Fast Flow (GE) and washed three times with wash buffer (20 mM Tris-HCl (pH 7.5), 10 mM MgCl_2_), suspended in kinase buffer (20 mM Tris-HCl (pH 7.5), 10 mM MgCl_2_, 100 μM ATP) supplemented with the phosphatase inhibitor cocktail EDTA free (Nacalai tesque). The His-PLK1-bound beads were mixed with the GST-fused proteins and incubated at 30°C for 30 minutes. After the kinase reaction the supernatant was collected by centrifugation, reactions were stopped by boiling in 2×Laemmli sample buffer supplemented with 10% β-mercaptoethanol. SDS-PAGE and transfer to PDVF membrane (Millipore) were performed by standard methods. To detect phosphorylations, the membrane was incubated for 1 hour with Phos-tagTM BLT-104 (NARD Institute) prepared according to the manufacturer’s instructions and then probed with Streptavidin-conjugate HRP (Jackson). To detect the GST-fusion proteins, the membrane blocked with Blocking One (nacalai tesque) was incubated for 30 minutes with GST-tag HRP-DirecT (MBL) antibody. The HRP signals were detected using Luminata Forte Western HRP Substrate (Millipore) by LAS 3000 mini (GE).

## Supporting Information

S1 Fig PLK1 localizations in live oocytes. (A) Time-lapse imaging of meiosis I in oocytes expressing EGFP-PLK1 (green) and H2B-mCherry (chromosomes, red). Maximum intensity z-projection images at representative time points are shown. Time after NEBD (h:mm). Scale bar = 10 μm. (B) The mean intensities of EGFP-PLK1 at 10 kinetochores and 5 MTOCs selected on images shown in Fig. 1A at each timepoint (prometaphase, 2 hours after NEBD; metaphase, 5 hours after NEBD), and at the anaphase spindle midzone were measured. The data were normalized by the value of the prometaphase kinetochores. Average and s.d. are shown (n = 3 oocytes). (C) The mean intensities of BUBR1 pT669 (green) and BUBR1 (red) at 20 kinetochores selected from each BI2536 concentration were measured. The BUBR1 pT669 level relative to BUBR1 was calculated and normalized by the value of the 0 nM. Average and s.d. are shown (n = 5 oocytes at 3 hours after NEBD). ***p < 0.0001. (D) The mean intensities of histone H3 pS28 and histone H3 at chromosomes were measured. The H3 pS28 level relative to H3 was calculated and normalized by the value of the 0 nM. Average and s.d. are shown (n = 5 oocytes at 3 hours after NEBD). (E) The mean intensities of EGFP-PLK1 at 10 kinetochores and 10 MTOCs were measured. The data were normalized by the value of the control kinetochores and control MTOCs. Average and s.d. are shown (n = 3 oocytes at 6 hours after NEBD). *p < 0.05, ** p < 0.01.
(TIF)

S2 Fig Analysis of chromosome condensation and NEBD in BI2536- and flavopiridol-treated oocytes. Determination of the onset and end of 70 kDa dextran influx and the onset and end of chromosome condensation from curves from individual oocytes. Dextran signals were normalized by scaling between 0 and 1 according global minimum and maximum, and chromosome volume was normalized to 1 as described in Fig. 2C.
(TIF)

S3 Fig PLK1 does not control lamin disassembly. (A) Time lapse imaging of lamin B1-EGFP and H2B-mCherry in oocytes after induction of meiotic resumption in control, 100 nM BI2536 and 1 μM flavopiridol medium. Pictures represent single section from bright field (BF), single confocal section of lamin B1-EGFP (green) and maximum intensity z-projection for H2B-mCherry (red). Scale bar = 20 μm. (B) Length of lamin B1-EGFP disassembly. Means with 95% confidence intervals are presented (n = 16, 21, 12; ***p < 0.0001).
(TIF)

S4 Fig Smaller spindles are formed in PLK1-inhibited oocytes. (A) Volume rendering of the signals of EGFP-MAP4 (microtubules, green) and H2B-mCherry (chromosomes, red) in the presence of DMSO (control) or 100 nM BI2536. (B) The volume of the spindle was measured throughout meiosis I. Time after NEBD (h). Average and s.d. are shown (n = 8, 17).
(TIF)

S5 Fig PLK1 is required for chromosome alignment. (A) Kinetochore positions were determined from the time-lapse images of EGFP-CENP-C and H2B-mCherry in oocytes cultured in the presence of DMSO (control) or 100 nM BI2536. The kinetochore positions are shown in the 3D plot as green spheres. Red bars connect homologous kinetochores. The view perpendicular to the chromosome distribution equator (top view) is shown. Time after NEBD (h:mm). The unit of the grid is 5 μm. (B) Chromosome distribution was viewed perpendicular to the equator, and the distance between the center and the chromosome was measured. The values were normalized by the distance of the most distal chromosome. Chromosomes that show >0.707 normalized distances were categorized as located in the prometaphase belt region, and its fraction was plotted over time. The time of prometaphase belt formation was defined as the time when the number of chromosomes that were located in the prometaphase belt region reached a maximum. Data from three oocytes in each condition are shown. Time after NEBD (h). (C) Distance between the paired kinetochores of homologous chromosomes was measured. Average and s.d. are plotted (n = 60, 60 from three oocytes cultured in each condition. ***p < 0.001).
(TIF)

S6 Fig EMI1 is phosphorylated by PLK1 *in vitro*. Recombinant GST-EMI1 or GST–EMI1–2A (S124A and S128A) proteins were incubated with His-PLK1-bound beads and analyzed the phosphorylation level with Phos-tag. The signals were measured, and the phosphorylation level relative to the protein level was measured and normalized.
(TIF)

S7 Fig Cyclin B is destroyed normally when BI2536 is added after MG132 release. The same experimental scheme as in Fig. 7A was applied, but cyclin B-EGFP was expressed.(A) Anaphase phenotypes after MG132 release in control and BI2536-treated oocytes expressing cyclin B-EGFP. PB = polar body. Note that the BI2536-treated oocytes did not exhibit DNA decondensation. Compare with Fig. 7B. (B) Time-lapse imaging of cyclin B-EGFP (green) and H2B-mCherry (red) after DMSO (control, top) or 100 nM BI2536 (bottom) was added at the time of the MG132 release (h:mm). Each phenotype from S7A Fig. is shown on a representative image sequence. Scale bar = 20 μm. (C) Quantification of cyclin B-EGFP destruction. Values were normalized to 1 at the time when imaging was started. Time relative to MG132 washout. The “BI2536 with anaphase” curve represents BI2536-treated oocytes that underwent abnormal chromosome segregation (3^rd^ row in S8B Fig.). The “BI2536 w/o anaphase” curve represents BI2536-treated oocytes that did not undergo chromosome segregation (2^nd^ row in S8B Fig.). Average and s.d. are shown (n = 11, 12). (D) Degradation rate of cyclin B-EGFP was calculated from (C). Average and s.d. are shown.
(TIF)

S1 Movie PLK1 localizes to MTOCs, kinetochores and the spindle midzone. Time-lapse imaging of meiosis I in oocytes expressing EGFP-PLK1 (green) and 3mCherry-CENP-C (kinetochores, red). Maximum intensity z-projection images are shown. Color-merged (left) and grayscale for EGFP-PLK1 (right). Time after induction of meiotic resumption (hh:mm:ss). Scale bar = 10 μm.
(MP4)

S2 Movie Chromosome condensation and nuclear envelope permeabilization in BI2536- and flavopiridol-treated oocytes. The green channel represents the maximum intensity z-projection of H2B-EGFP signal, red channel represents single confocal section of 70-kDa-dextran-TAMRA signal. Time after induction of meiotic resumption (minutes).
(MP4)

S3 Movie PLK1 is required for efficient formation of the acentriolar spindle. Time-lapse imaging of meiosis I in oocytes expressing EGFP-MAP4 (microtubules, green) and H2B-mCherry (chromosomes, red) in the presence of DMSO (control, left) or 100 nM BI2536 (right). Maximum intensity z-projection images are shown. Time after NEBD (hh:mm:ss). Scale bar = 10 μm.
(MP4)

S4 Movie Complete kinetochore tracking during meiosis I in PLK1-inhibited oocytes. Time-lapse imaging of meiosis I in oocytes expressing EGFP-CENP-C (kinetochores, green) and H2B-mCherry (chromosomes, red) in the presence of DMSO (control, left) or 100 nM BI2536 (right). Maximum intensity z-projection images are shown. White lines indicate kinetochore tracks over 5 timepoints. Time after NEBD (hh:mm:ss). Scale bar = 10 μm.
(MP4)

S5 Movie PLK1 activates the APC/C through multiple pathways. Time-lapse imaging of oocytes expressing securin-EGFP (green) and H2B-mCherry (chromosomes, red) in the presence of DMSO (control, top left), 100 nM BI2536 (top right), 1 μM reversine (bottom left), or both of BI2536 and reversine (bottom right). Maximum intensity z-projection images are shown. Time after NEBD (h). Scale bar = 10 μm.
(MP4)

S6 Movie PLK1 is required for full activation of the APC/C. Time-lapse imaging of securin-EGFP (green) and H2B-mCherry (red) after DMSO (control, top) or 100 nM BI2536 (bottom) was added at the time of metaphase I (6.5 hours after the induction of meiotic resumption). Time after BI2536 addition (hh:mm). Scale bar = 20 μm.
(MP4)

S7 Movie PLK1 is essential for chromosome segregation, first polar body extrusion, and maintenance of the condensed state of chromosomes. Time-lapse imaging of securin-EGFP (green) and H2B-mCherry (red) after DMSO (control, top left) or 100 nM BI2536 was added at the time of the MG132 washout (h). The BI2536-treated oocytes that exhibited no chromosome segregation (top right), abnormal chromosome segregation (bottom left), and chromosome decondensation after abnormal segregation (bottom right) are shown. Time after MG132 washout (hh:mm). Scale bar = 20 μm.
(MP4)
