# Supplementary figures and images for: Multiple Requirements of PLK1 during Mouse Oocyte Maturation
Source: PLoS One. 2015 Feb 6;10(2):e0116783. doi: 10.1371/journal.pone.0116783 (PMC4319955; doi:10.1371/journal.pone.0116783)

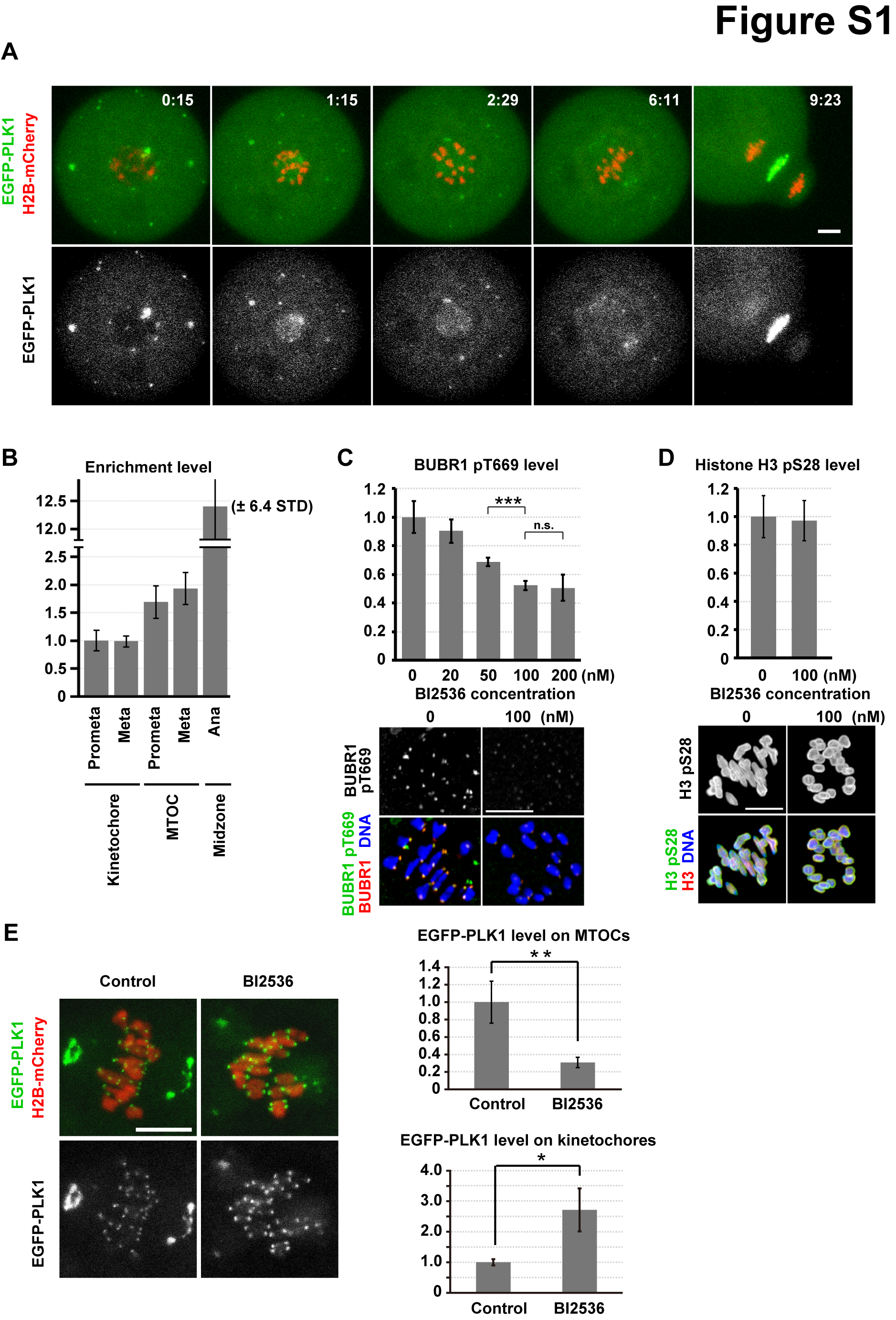

Supplement: S1 Fig — (A) Time-lapse imaging of meiosis I in oocytes expressing EGFP-PLK1 (green) and H2B-mCherry (chromosomes, red). Maximum intensity z-projection images at representative time points are shown. Time after NEBD (h:mm). Scale bar = 10 μm. (B) The mean intensities of EGFP-PLK1 at 10 kinetochores and 5 MTOCs selected on images shown in Fig. 1A at each timepoint (prometaphase, 2 hours after NEBD; metaphase, 5 hours after NEBD), and at the anaphase spindle midzone were measured. The data were normalized by the value of the prometaphase kinetochores. Average and s.d. are shown (n = 3 oocytes). (C) The mean intensities of BUBR1 pT669 (green) and BUBR1 (red) at 20 kinetochores selected from each BI2536 concentration were measured. The BUBR1 pT669 level relative to BUBR1 was calculated and normalized by the value of the 0 nM. Average and s.d. are shown (n = 5 oocytes at 3 hours after NEBD). ***p < 0.0001. (D) The mean intensities of histone H3 pS28 and histone H3 at chromosomes were measured. The H3 pS28 level relative to H3 was calculated and normalized by the value of the 0 nM. Average and s.d. are shown (n = 5 oocytes at 3 hours after NEBD). (E) The mean intensities of EGFP-PLK1 at 10 kinetochores and 10 MTOCs were measured. The data were normalized by the value of the control kinetochores and control MTOCs. Average and s.d. are shown (n = 3 oocytes at 6 hours after NEBD). *p < 0.05, ** p < 0.01. (TIF) [file pone.0116783.s001.tif]

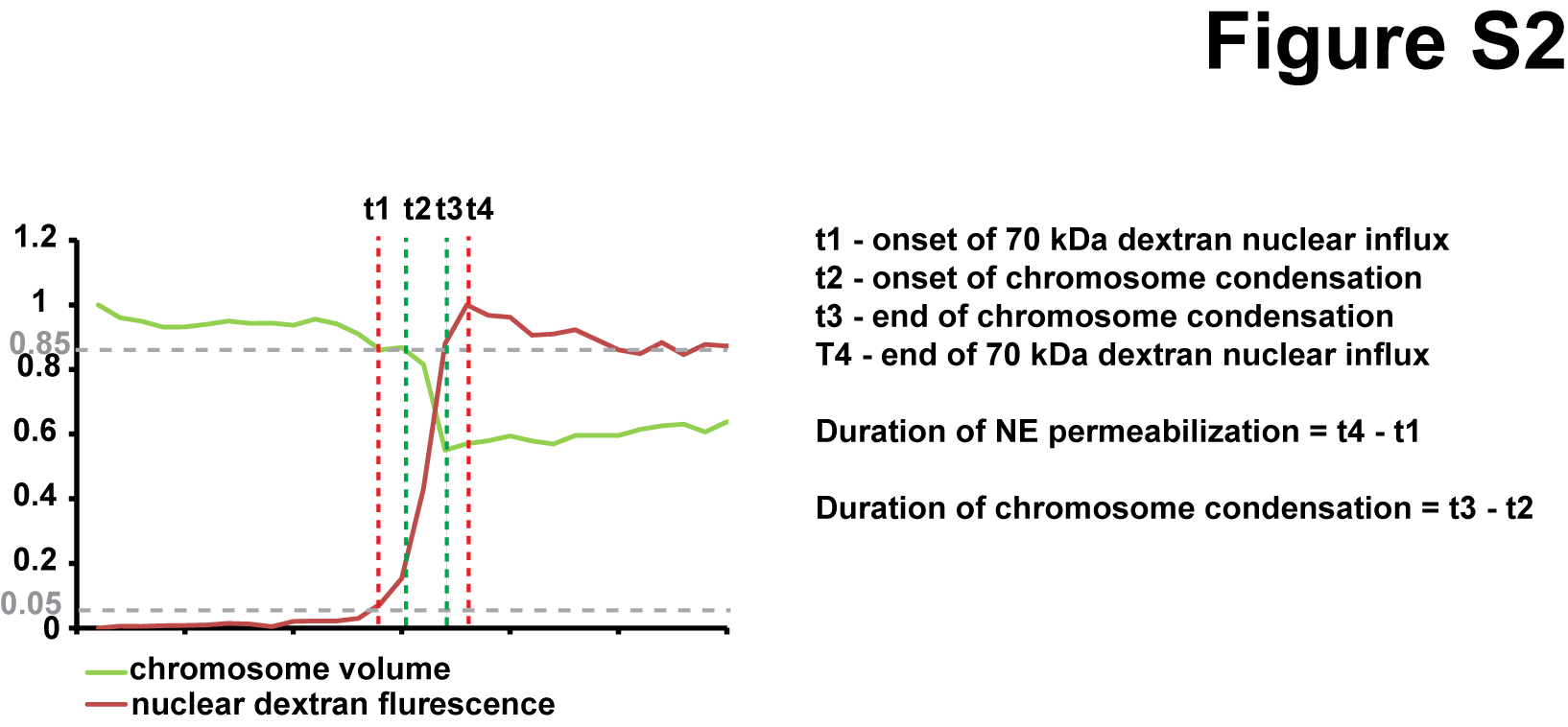

Supplement: S2 Fig — Determination of the onset and end of 70 kDa dextran influx and the onset and end of chromosome condensation from curves from individual oocytes. Dextran signals were normalized by scaling between 0 and 1 according global minimum and maximum, and chromosome volume was normalized to 1 as described in Fig. 2C. (TIF) [file pone.0116783.s002.tif]

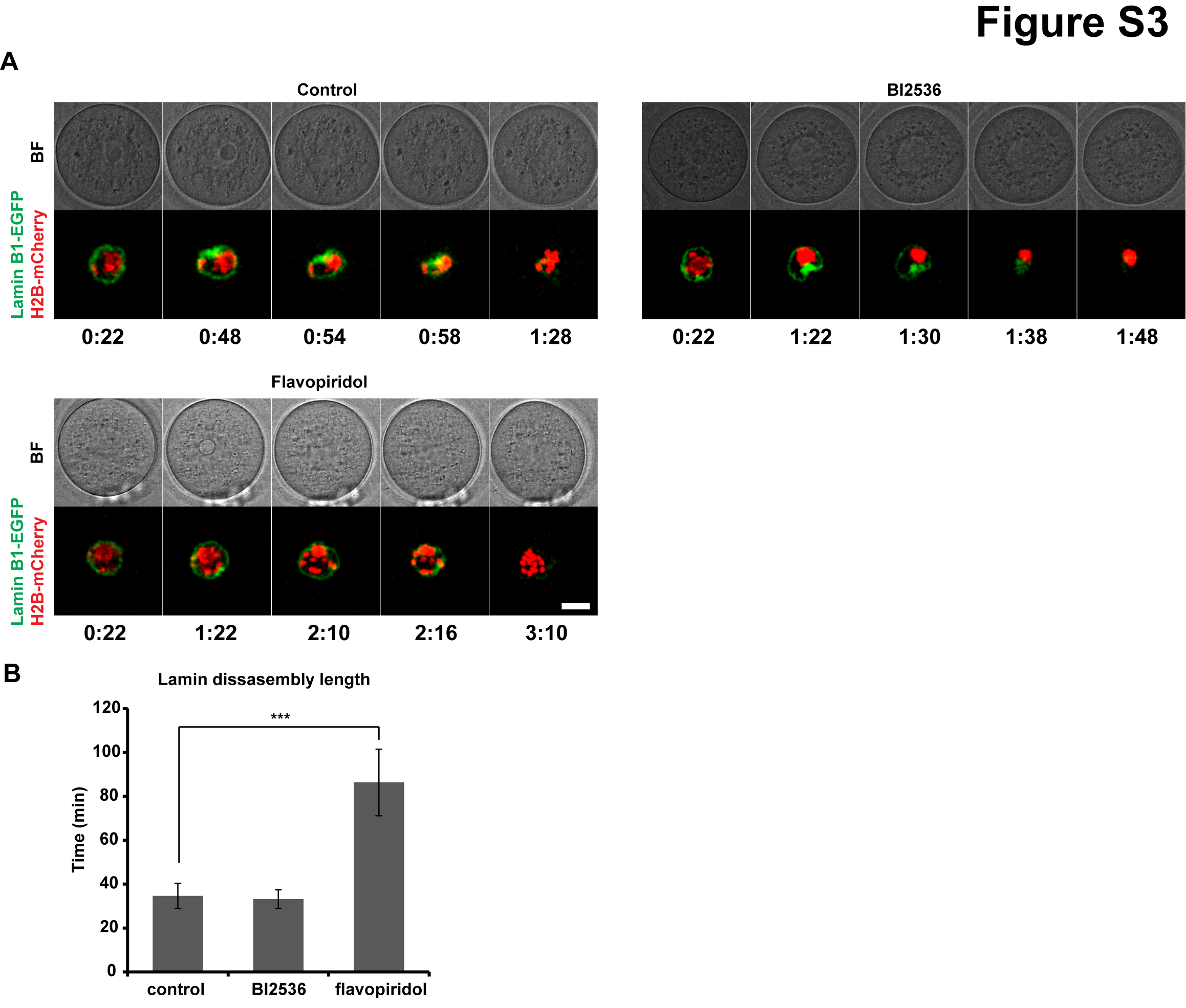

Supplement: S3 Fig — (A) Time lapse imaging of lamin B1-EGFP and H2B-mCherry in oocytes after induction of meiotic resumption in control, 100 nM BI2536 and 1 μM flavopiridol medium. Pictures represent single section from bright field (BF), single confocal section of lamin B1-EGFP (green) and maximum intensity z-projection for H2B-mCherry (red). Scale bar = 20 μm. (B) Length of lamin B1-EGFP disassembly. Means with 95% confidence intervals are presented (n = 16, 21, 12; ***p < 0.0001). (TIF) [file pone.0116783.s003.tif]

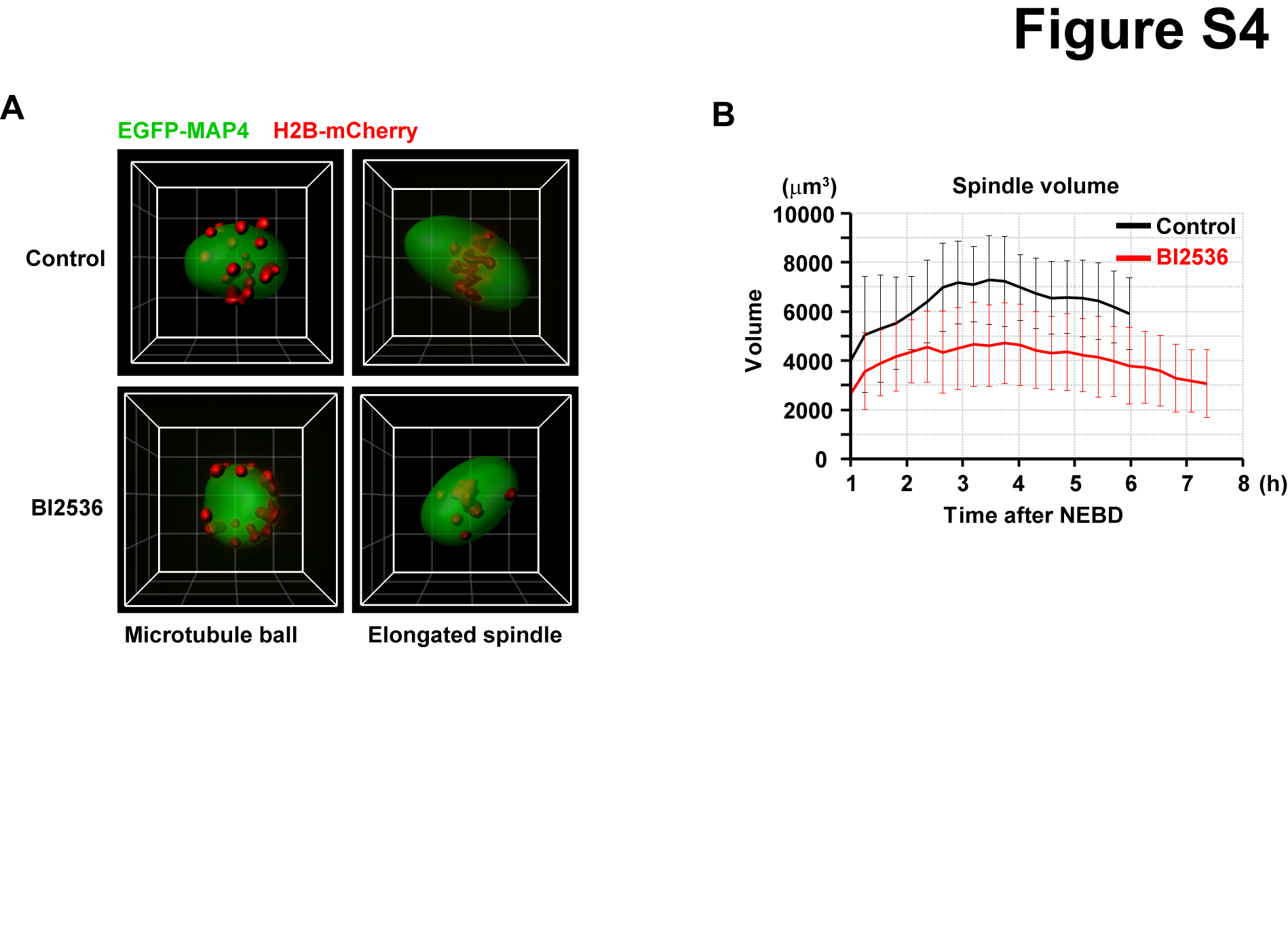

Supplement: S4 Fig — (A) Volume rendering of the signals of EGFP-MAP4 (microtubules, green) and H2B-mCherry (chromosomes, red) in the presence of DMSO (control) or 100 nM BI2536. (B) The volume of the spindle was measured throughout meiosis I. Time after NEBD (h). Average and s.d. are shown (n = 8, 17). (TIF) [file pone.0116783.s004.tif]

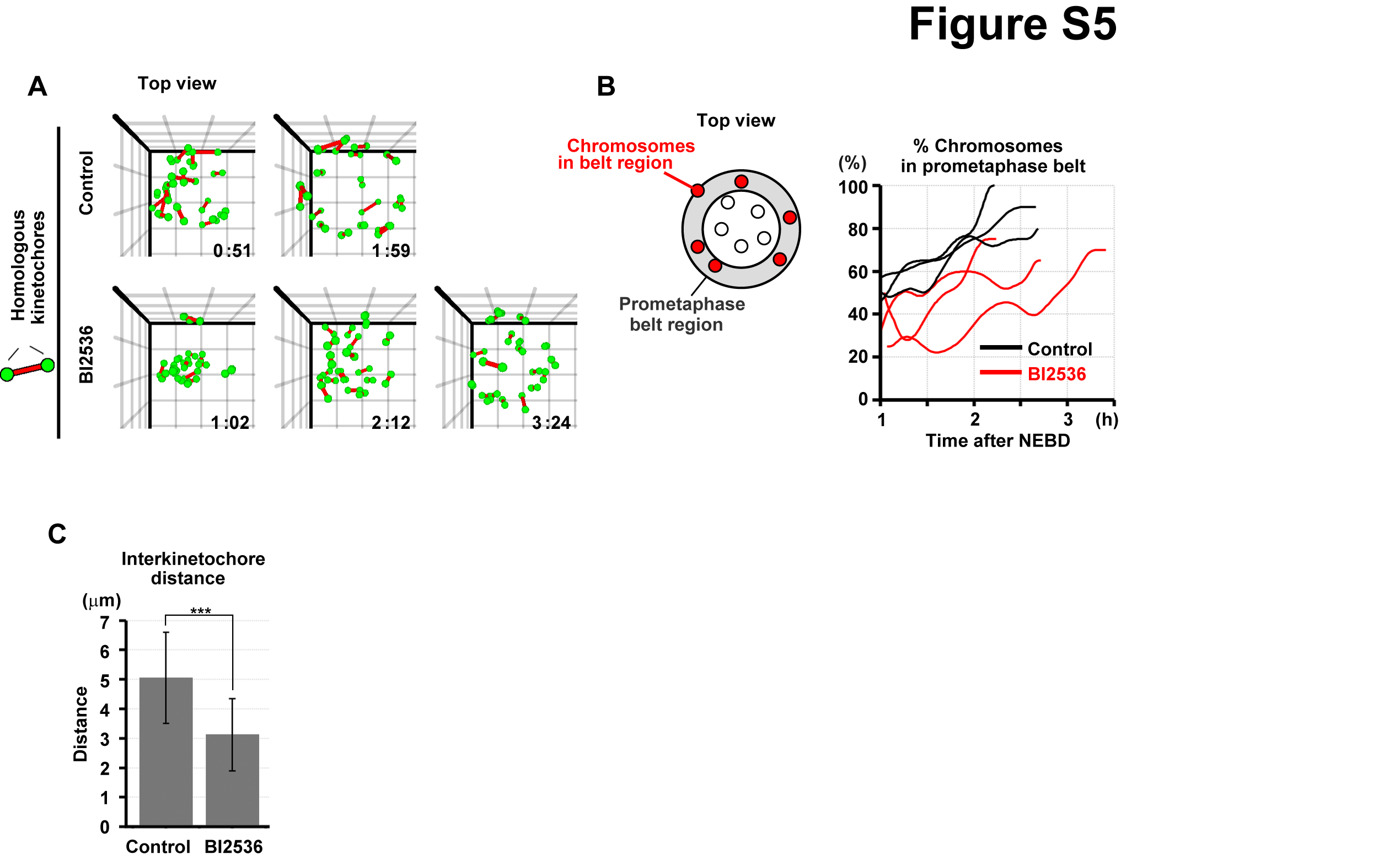

Supplement: S5 Fig — (A) Kinetochore positions were determined from the time-lapse images of EGFP-CENP-C and H2B-mCherry in oocytes cultured in the presence of DMSO (control) or 100 nM BI2536. The kinetochore positions are shown in the 3D plot as green spheres. Red bars connect homologous kinetochores. The view perpendicular to the chromosome distribution equator (top view) is shown. Time after NEBD (h:mm). The unit of the grid is 5 μm. (B) Chromosome distribution was viewed perpendicular to the equator, and the distance between the center and the chromosome was measured. The values were normalized by the distance of the most distal chromosome. Chromosomes that show >0.707 normalized distances were categorized as located in the prometaphase belt region, and its fraction was plotted over time. The time of prometaphase belt formation was defined as the time when the number of chromosomes that were located in the prometaphase belt region reached a maximum. Data from three oocytes in each condition are shown. Time after NEBD (h). (C) Distance between the paired kinetochores of homologous chromosomes was measured. Average and s.d. are plotted (n = 60, 60 from three oocytes cultured in each condition. ***p < 0.001). (TIF) [file pone.0116783.s005.tif]

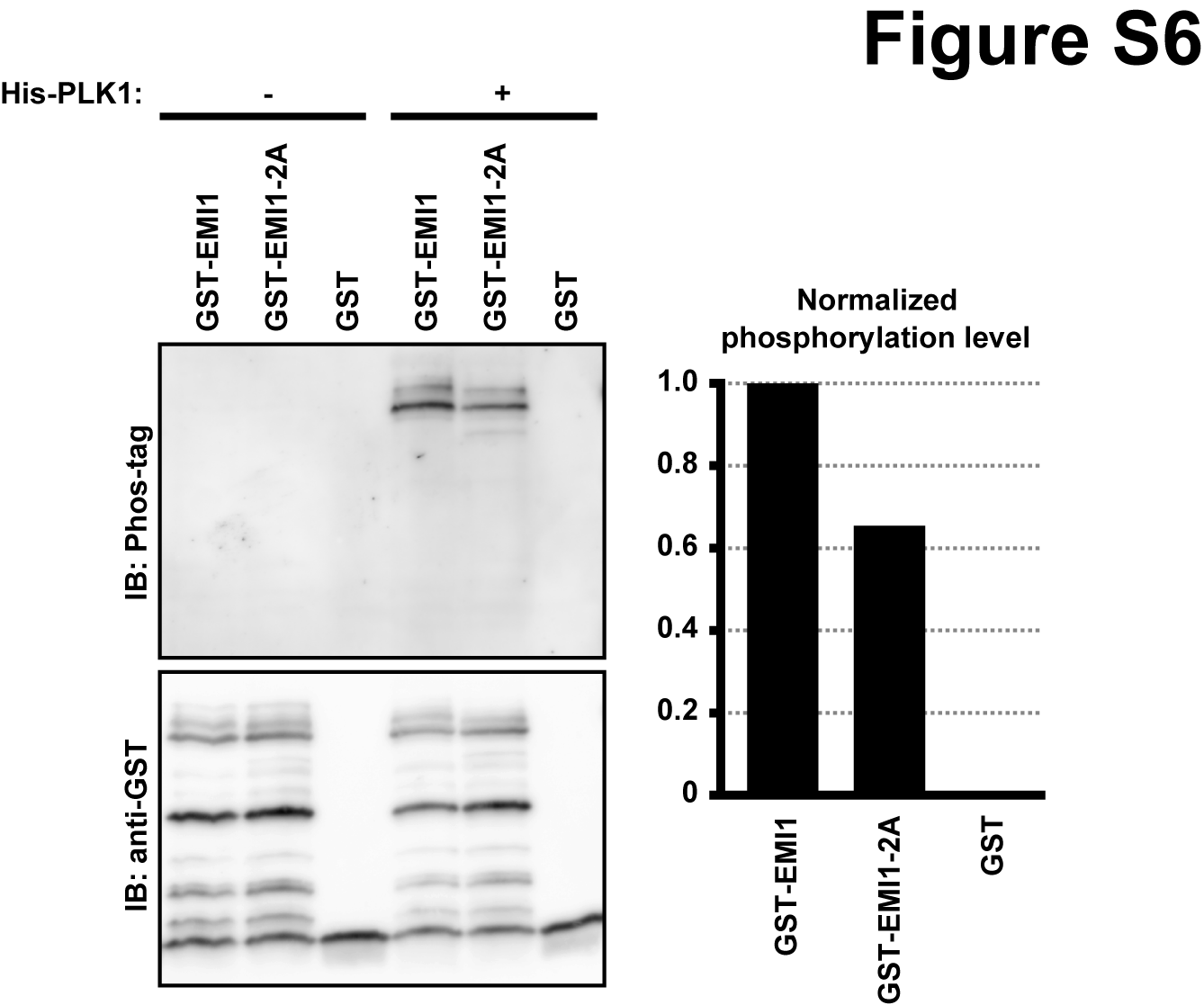

Supplement: S6 Fig — Recombinant GST-EMI1 or GST–EMI1–2A (S124A and S128A) proteins were incubated with His-PLK1-bound beads and analyzed the phosphorylation level with Phos-tag. The signals were measured, and the phosphorylation level relative to the protein level was measured and normalized. (TIF) [file pone.0116783.s006.tif]

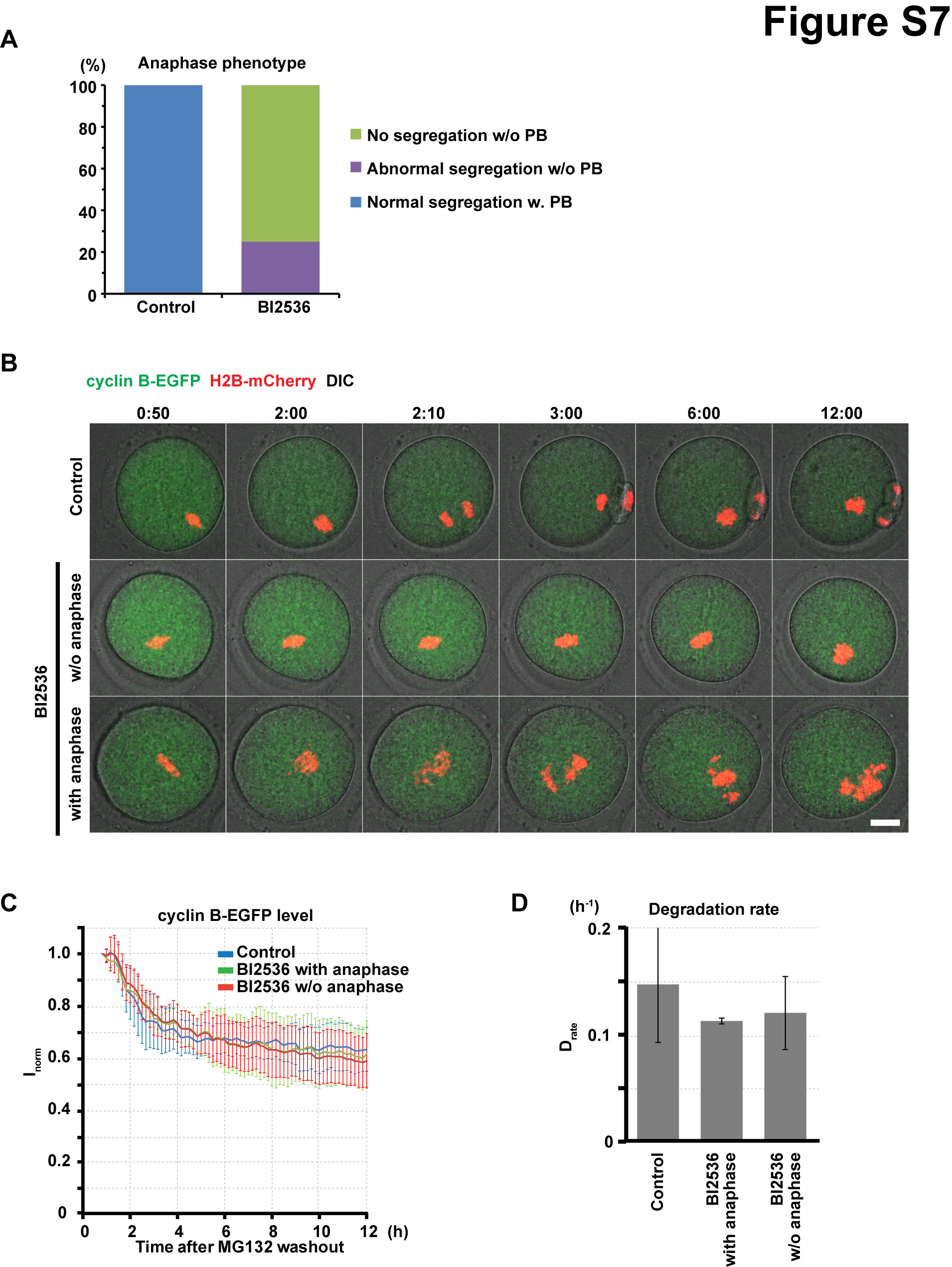

Supplement: S7 Fig — The same experimental scheme as in Fig. 7A was applied, but cyclin B-EGFP was expressed.(A) Anaphase phenotypes after MG132 release in control and BI2536-treated oocytes expressing cyclin B-EGFP. PB = polar body. Note that the BI2536-treated oocytes did not exhibit DNA decondensation. Compare with Fig. 7B. (B) Time-lapse imaging of cyclin B-EGFP (green) and H2B-mCherry (red) after DMSO (control, top) or 100 nM BI2536 (bottom) was added at the time of the MG132 release (h:mm). Each phenotype from S7A Fig. is shown on a representative image sequence. Scale bar = 20 μm. (C) Quantification of cyclin B-EGFP destruction. Values were normalized to 1 at the time when imaging was started. Time relative to MG132 washout. The “BI2536 with anaphase” curve represents BI2536-treated oocytes that underwent abnormal chromosome segregation (3rd row in S8B Fig.). The “BI2536 w/o anaphase” curve represents BI2536-treated oocytes that did not undergo chromosome segregation (2nd row in S8B Fig.). Average and s.d. are shown (n = 11, 12). (D) Degradation rate of cyclin B-EGFP was calculated from (C). Average and s.d. are shown. (TIF) [file pone.0116783.s007.tif]
